# The effects of shockwave therapy on musculoskeletal conditions based on changes in imaging: a systematic review and meta-analysis with meta-regression

**DOI:** 10.1186/s12891-020-03270-w

**Published:** 2020-04-28

**Authors:** Hani Al-Abbad, Sophie Allen, Susan Morris, Jackie Reznik, Erik Biros, Bruce Paulik, Anthony Wright

**Affiliations:** 1 School of Physiotherapy and Exercise Science, Curtin University, GPO Box U1987, Perth, WA Australia; 2 Physical Therapy department, Rehabilitation hospital, King Fahad Medical City, Riyadh, Saudi Arabia; 3 College of Healthcare Science and Division of Tropical Health and Medicine, James Cook University, Townsville, QLD Australia; 4 College of Medicine and Dentistry, James Cook University, Townsville, Australia; 5 HFRC, Nedlands, WA Australia

**Keywords:** Extracorporeal shockwave therapy, Imaging, Systematic review, Meta-analysis

## Abstract

**Background:**

Shockwave therapy (SWT) is a commonly used intervention for a number of musculoskeletal conditions with varying clinical outcomes. However, the capacity of SWT to influence pathophysiological processes and the morphology of affected tissues remains unclear. The objective of the current review is to evaluate changes in imaging outcomes of musculoskeletal conditions following SWT.

**Methods:**

A comprehensive search of Medline, Embase, Cochrane Controlled Trials Register, CINAHL and SportDiscus was conducted from inception to October 2018. Prospective clinical trials evaluating the effectiveness of SWT based on changes in imaging outcomes were eligible for inclusion. Articles were evaluated independently for risk of bias using the Cochrane Risk of Bias list and the Methodological Index for Non-Randomized Studies. Random-effects meta-analysis and meta-regression with a priori determined covariates was conducted for each condition to determine potential predictors of SWT effects.

**Results:**

Sixty-three studies were included, with data from 27 studies available for effect size pooling. Meta-analyses and meta-regression on imaging outcomes were performed for rotator cuff calcific tendinitis (*n* = 11), plantar fasciitis (*n* = 7) and osteonecrosis of the femoral head (*n* = 9). There was an overall reduction in the size of measured lesion following SWT (MD 8.44 mm (95%CI -4.30, 12.57), *p* < 0.001) for calcium deposit diameter, (MD 0.92 mm (95%CI -0.03, 1.81), *p* = 0.04) for plantar fascia thickness and (MD 4.84% (95%CI -0.06, 9.75), *p* = 0.05) for lesion size in femoral head osteonecrosis. Meta-regression showed no influence of SWT dosage parameters, however, baseline lesion size was an independent predictor for changes in imaging outcomes.

**Conclusions:**

SWT altered the morphology of musculoskeletal conditions, potentially reflecting changes in underlying pathophysiological processes. The parameters of SWT dosage are not significant predictors of changes in imaging outcomes. Lack of adequate reporting of imaging outcomes limited the conclusions that could be drawn from the current review. Registration number: PROSPERO CRD42018091140.

## Background

Shockwave Therapy (SWT) is used to treat a range of musculoskeletal conditions. Focused and radial shockwave (radial pressure waves) are two technically distinct forms of SWT. It has been argued that focused shockwave therapy and radial shockwave therapy should be viewed as distinctly different therapeutic modalities [1]. However, despite the differences in their physical characteristics, method of energy generation and shockwave propagation, focused and radial shockwave types share common clinical indications [2]. SWT is often indicated as a secondary conservative treatment choice for recalcitrant musculoskeletal conditions, unresponsive to standard care [1, 3]. These indications include plantar fasciitis, Achilles tendinopathy, patellar tendinopathy, calcific and non-calcific shoulder tendinopathy and lateral epicondylitis. Also, bone and cartilage related disorders such as non-union of fractures, osteonecrosis of the femoral head and knee osteoarthritis related bone marrow edema (BME) are among the range of SWT clinical indications. Research evidence for SWT clinical effectiveness varies across the indicated conditions. Good evidence based on systematic reviews exists to support the use of SWT for calcific tendinopathy of the shoulder [4], Achilles tendinopathy [5, 6], knee osteoarthritis [7], early stage osteonecrosis of the femoral head [8] and plantar fasciitis [9]. However, research evidence for the effectiveness of SWT in lateral epicondylitis is variable [1] and is lacking for patellar tendinopathy [3, 6].

Focused shockwaves are generated through three mechanisms: electrohydraulic, piezoelectric or electromagnetic methods that convert electrical energy into kinetic energy, whilst radial shockwaves are generated pneumatically [2]. The proposed mechanism of action for SWT is based on mechano-transduction [10]. The delivery of mechanical acoustic energy to the target tissue induces molecular, cellular and tissue responses [11]. Based on animal studies, SWT promotes the expression of various angiogenic and osteogenic growth factors such as vascular endothelial growth factor (VEGF) and bone morphogenetic protein (BMP), which promotes tissue regeneration [12]. In addition, SWT has an anti-inflammatory effect by modulating the expression of interleukins (IL-6 and IL-10) and other cytokines [13].

Clinical outcomes such as pain rating and functional disability scores are commonly used to evaluate the effectiveness of SWT. However, the utilization of objective outcome measures that evaluate changes in the affected musculoskeletal tissues is required to provide evidence of SWT influence on pathophysiological processes in humans. Different imaging techniques are used clinically to establish a diagnosis, guide the delivery of an intervention or to evaluate the effectiveness of an intervention. A range of imaging modalities including magnetic resonance imaging (MRI), ultrasonography (US), Computed Tomography (CT), dual-energy x-ray absorptiometry (DEXA) or plain radiography have been used to support diagnosis and in some cases to evaluate outcomes in studies investigating SWT. Despite lack of consistency in utilization, these imaging modalities are valuable tools for evaluating the morphological characteristics of injured tissues. That can then be used clinically to monitor improvements in the underlying pathophysiological process following an intervention. Therefore, the utilization of suitable imaging tools at appropriate time points as an adjunct to clinical examination may provide a better understanding of the tissue pathophysiology and support the management planning process. Little is known about the capacity of SWT to induce positive improvements in pathophysiological processes in musculoskeletal disorders, as indicated by changes in imaging parameters and no systematic reviews have been conducted on this topic. Therefore, the primary aim of this review was to explore the available evidence from clinical prospective trials with regard to any changes in the morphology of musculoskeletal lesions following SWT, as measured by imaging parameters. The secondary aim was to investigate significant predictors for the SWT effects using meta-regression. We also sought to make recommendations for future research studies.

## Methods

### Protocol and registration

This systematic review and meta-analysis was conducted following the guidelines of the Preferred Reporting Items for Systematic Reviews and Meta-analyses (PRISMA). The protocol of the current review was registered on the International Prospective Register of Systematic Reviews (PROSPERO; 2018, CRD42018091140).

### Search strategy

A comprehensive search using the electronic databases of MEDLINE, EMBASE, CINAHL, SPORTDiscuss and the Cochrane Controlled Trials Register using the PICOS format (population, intervention/exposure, comparison, outcome and study design) was conducted in October 2018. A range of keywords integrated with subject headings relevant to the review were systematically searched to maximize the search results. Keywords for the population category contained terms related to musculoskeletal conditions such as arthritis, fractures, bone marrow edema and tendinopathies. The intervention category contained keywords related to shockwave therapy. The outcome category contained terms related to different imaging methods (e.g. X-ray, MRI, Ultrasound imaging). No specific comparator was added as the primary aim of the review was to examine post intervention imaging changes. An example of the basic search strategy for databases is presented in Additional file 1.

Eligibility criteria included prospective study designs to avoid any potential selection bias inherent in retrospective studies, adult participants of both genders with an established musculoskeletal diagnosis and reported pre and post imaging measures to facilitate evaluation of changes in affected tissue morphology following SWT of any type (focused and radial). Non-human and non-English language studies were excluded.

### Study identification

The principal author conducted the database search. Study screening was carried out using the Covidence platform [14]. Two independent reviewers (HA and SA) screened titles and abstracts to determine eligibility for inclusion. The decision to include or exclude studies, based on the eligibility criteria, was made independently by each author. Full-texts of potentially relevant studies were retrieved for further assessment. Discrepancies in opinions were resolved by a third senior reviewer (AW).

### Risk of bias assessment

The quality of studies was independently evaluated by two reviewers (HA and SA). A third senior reviewer (AW) was involved to resolve differences in assessment. Randomized Controlled Trials (RCTs) were evaluated using the Cochrane Risk of Bias list [15]. The tool evaluates the risk of bias in five domains (selection, performance, attrition, reporting and other) for seven elements to be judged as high, unclear or low risk. A three-point scale was used to assign each degree of risk a number value with higher numbers indicating a lower risk of bias (low risk = 2, unclear risk = 1 and high risk = 0). The maximum score of 14 indicates the lowest risk of bias for a given study.

The Methodological Index for Non-Randomized Studies (MINORS) tool [16, 17] was used to evaluate the non-randomized studies. The MINORS is a valid and reliable tool that contains 12 methodological items, the first eight items measure attributes specific to non-comparative studies. Four additional items are evaluated only for studies with comparative groups. A higher score indicates a lower risk of bias.

The risk of bias was assessed mainly at the reported measured imaging outcomes level. The score for each risk of bias tool was divided by the total number of points possible to calculate the risk of bias percentage for each study.

### Data extraction

One reviewer (HA) extracted relevant data from individual studies and the data were independently checked by a second reviewer (SA) for consistency. Extracted data included study characteristics such as study design, participant numbers and demographics, the condition treated, parameters and dosage of the SWT intervention (level of energy, number of shocks and sessions), follow-up period, imaging modality description and findings. Authors were contacted when relevant outcomes were incompletely reported and were asked to provide missing information. When the values of standard deviations (SD) were not provided for the intervention or control group, these were calculated from confidence intervals, and *p*-values for differences in means, or for group means using the RevMan calculator [18].

### Primary outcome

Changes in measures derived from imaging methods such as MRI, ultrasonography, CT, DEXA or plain radiography reflecting morphological changes in affected musculoskeletal tissues following SWT were the primary evaluated outcomes for this review. The measures of effect were pre to post-imaging changes, demonstrating presence, grade, signal intensity or size of the tissue lesion. The strategy for data synthesis employed a quantitative method in the form of meta-analysis depending upon the type of data extracted, alongside narrative qualitative synthesis. The method of evaluation for each study is clearly stated in the relevant tables.

### Statistical analysis

Quantitative data analysis was performed using RevMan [18]. Meta-analysis was conducted to calculate pooled effect size for included studies. Continuous data were presented as mean differences (MD) and dichotomous data with odds ratios (OR), including the corresponding 95% confidence interval (CI). Pooling was performed using a random effects model to provide summary effect size owing to expected clinical and methodological heterogeneity. Subgroup analyses were performed separately for each musculoskeletal condition based on SWT type or dosage, control group or use of imaging guidance. Heterogeneity was assessed statistically with χ^2^ test and I^2^ statistics and significance was set at *p* < 0.05. Meta-regression was carried out to explain the source of heterogeneity based on important mediating covariates such as SWT dose parameters, baseline lesion size, utilization of anesthesia and imaging guidance (determined a priori).

## Results

### Study selection and characteristics

**Fig. 1:**
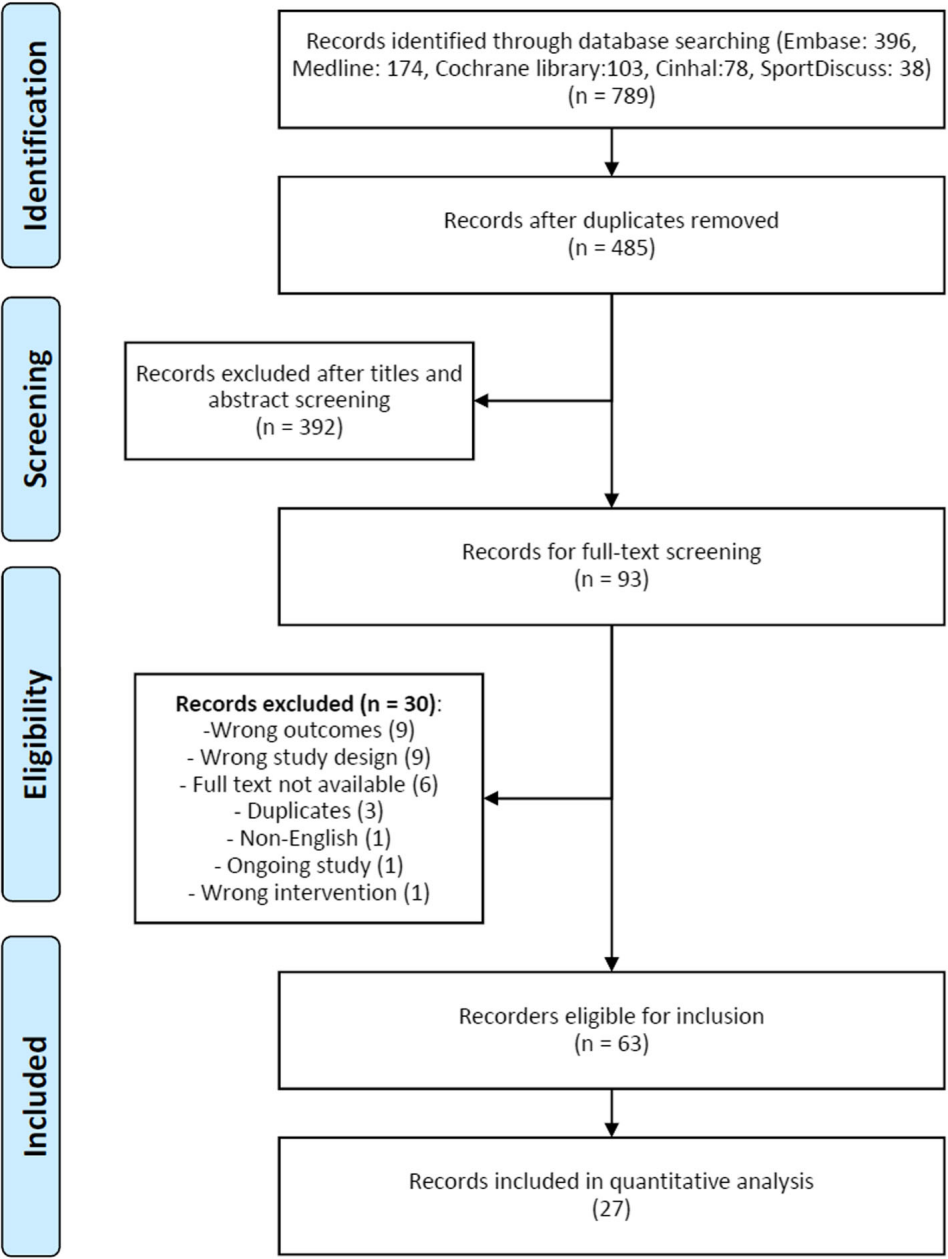
PRISMA flow chart of study selection process

The database searches resulted in 789 titles. (Fig. 1). Following the removal of duplicates and exclusion of records based on title and abstract screening, a total of 93 studies were available for full-text review. A total of 30 studies were further excluded with inappropriate study design (predominantly retrospective studies) or lack of imaging outcome as the most frequent reasons for exclusion, leaving a final selection of 63 studies meeting the inclusion criteria. Of the included studies, 30 used an RCT design and the remaining 33 studies were prospective cohort trials.

### Description of included studies

**Fig. 2:**
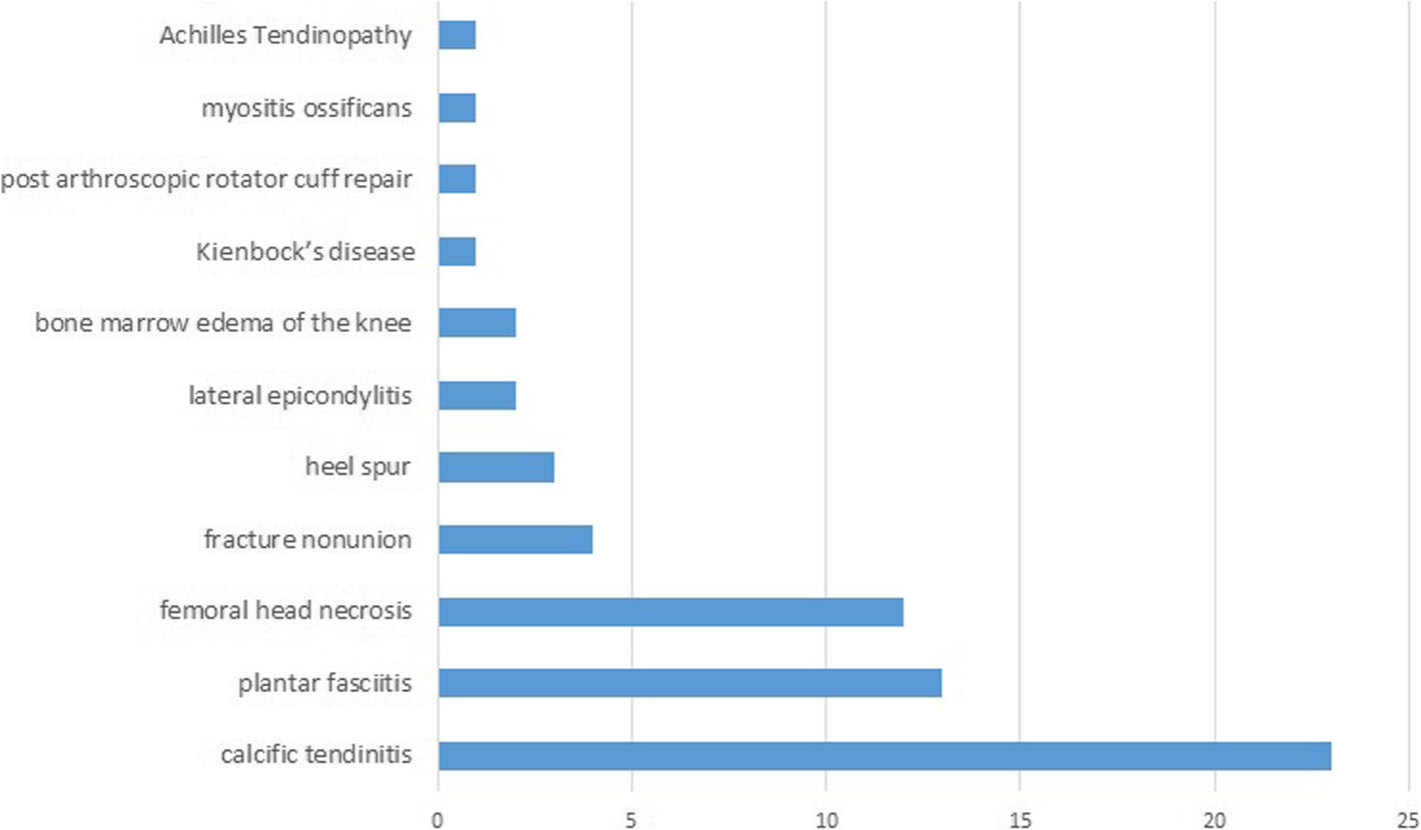
Musculoskeletal conditions of included studies

The total number of participants in the SWT groups (focused and radial SWT) was 3110. The most commonly evaluated musculoskeletal condition was rotator cuff calcific tendinitis (23 studies), followed by plantar fasciitis (13 studies), femoral head necrosis (12 studies) and fracture non-union (4 studies). There were a few other individual studies evaluating different musculoskeletal conditions (Fig. 2).

### Quality assessment

The risk of bias assessment is summarized in Additional file 2 for included RCTs and prospective trials respectively. The mean score (%) ± SD of the Cochrane risk of bias tool among the 31 RCT studies was 59.68 ± 18.95, while the mean score was 73.61 ± 7.65 for non-RCT studies according to the MINORS tool. Items of risk of bias rating across all included RCT and non-RCT studies are shown in Additional file 2. Inter-rater reliability of the risk of bias assessment was calculated using the kappa coefficient (k). The overall agreement between the two primary reviewers was 79.84% (k = 0.53) indicating moderate agreement.

### Rotator cuff calcific tendinitis

There were 23 studies (8 RCTs) published between 1995 and 2017 evaluating the effect of SWT on rotator cuff calcific tendinitis. The total sample was 1110 (1141 shoulders) participants. The main imaging inclusion criterion for participant recruitment was the presence of symptomatic type I or II calcification of the rotator cuff based on the Gartner and Simons radiographic classification [19], as detected on radiographs. Detection of glenohumeral or acromioclavicular arthritis, rotator cuff tear or type III calcification were the main exclusion criteria.

Focused type SWT was used in 21 studies, while two studies used radial type SWT. The mean ± SD SWT delivered shocks was 2104 ± 990.57 (1000–6000) and the mean energy flux density (EFD) was 0.26 ± 0.15 (0.02–0.6) mJ/mm^2^. The mean number of SWT sessions was 2.66 ± 1.91 (1–8) with 10.1 ± 7.26 (1–35) days interval between sessions. Ten studies used anesthesia prior to SWT application and 14 studies used imaging guidance to target calcium deposits. Further details on included studies characteristics and SWT parameters are presented in Table 1.

**Table 1:** Characteristics of studies and intervention details for rotator cuff calcifying tendinitis

Author (year)	Study design	N	Mean age ±SD or (range)	Mean symptoms duration ± SD or (range), months	Area of SWT application	Dosage in impulses*EFD (mJ/mm^**2**^)/bar	No. of sessions	Interval between sessions	Co- intervention, anesthesia
Albert (2007) [20]	RCT	40 each group	High SWT: 46.6 (31–64) Low SWT: 47.5 (32–69)	High SWT: 41.2 (6–120) Low SWT: 36.4 (7–160)	Calcific deposit was identified using fluoroscopy	High SWT: 2000*0.45 Low SWT: 2000*0.02	2	14 days	None, yes
Cacchio (2006) [21]	RCT	45	56.12 ± 1.98	14 ± 4.95	Seated with shoulder abducted at 45^o^ in external rotation, elbow at 90^o^, SWT was placed in the direction of calcifications.	2500*0.1, pressure of 2.5 bar	4	1 week	None
Charrin (2001) [22]	Prospective open design	32	49.8 ± 5.9	52.1 ± 48.5	Ultrasound was used to identify the lesions and aimed at the calcific deposit at all times.	2000*0.32	2 or 3	13.4 ± 6.4 days	None
Cosentino (2003) [23]	RCT	35	51.8	15 (10–20)	SWT was placed in the direction of the calcification based on sonographic examination	1200*0.28	4	4–7 days	None
Daecke (2002) [24]	Prospective comparative design	Group A: 56 Group B: 59	49 (28–77)	5 (1–36) years	SWT was performed after localization of the calcification in 2 planes by fluoroscopy	2000*0.3	Group A: 1 Group B: 2	1 week	None, yes
DeBoer (2017) [25]	RCT	14	53 (95%CI 48, 58)	> 6	NR	2500*0.1, pressure of 2.5 bar	5	1 week	None
DelCastillo-Gonzalez (2016) [26]	RCT	80	49 ± 7	NR	The calcification was identified by fluoroscopy in seated position	2000* 0.2	8	Twice weekly	None
Farr (2011) [27]	RCT	15	49.7 ± 9	> 6	The calcific deposit was located by fluoroscopy. The computer calculated angle and distance for maximum precision.	Group A: 3200*0.3 Group B: 1600*0.2	Group A: 1 Group B: 2	1 week	None, yes
Gerdesmeyer (2003) [28]	RCT	48 each	High: 51.6 ± 8.5 Low: 47.3 ± 8.5 placebo: 52.3 ± 9.8	High: 42.6 ± 23.2 Low: 42.8 ± 25.2 placebo: 41.3 ± 28.6	Using fluoroscopy in prone position as the shoulder was rotated until the calcific deposit was identified	High: 1500*0.32 Low: 6000*0.08	2	14 days	All groups received 10 physiotherapy sessions after SWT, no
Hsu (2008) [29]	RCT	33	54.4 (30–70)	12.3 (6–72)	NR	1000*0.55	2	14 days	None, yes
Jakobeit (2002) [30]		80	53.3	> 6	SWT was performed with retroversion and adduction of the shoulder as far as possible under ultrasound monitoring	1800*0.42	1–5	4–6 weeks	None, yes
Kim (2014) [31]	RCT	29	57.4 (47–78)	> 3	SWT was performed in the sitting position by aiming at the maximum sore spot according to anatomic targeting	1000*0.36	3	1 week	NSAIDs, no
Kransy (2005) [32]	RCT	40	49.4 (32.4–63.5)	30.5 (12–60)	In prone position, the calcific deposit had been positioned in the center of the scan unit.	2500*0.36	1	NA	None, yes
Loew (1999) [33]	RCT	20 each	46 (28–77)	36	The calcification was visualized using fluoroscopy before and at intervals during treatment.	Group 1: 2000*0.1 Group 2,3: 2000*0.3	Group 1,2: 1 Group 3: 2	1 week	None, yes
Lowe (1995) [34]	Prospective open design	20	50 (35–72)	> 12	SWT was performed as localization of the calcium deposit was achieved with an image intensifier that was adjusted automatically in two planes. The head was placed in a ventro-lateral position	2000*18–22 kV	2	2 weeks	None, yes
Moretti (2005) [35]	Prospective open design	54	43 (34–66)	> 3	NR	2500*0.11	4	3 days	None
Pan (2003) [36]	RCT	32	55.21 ± 2.01	24.55 ± 6.45	SWT was positioned at the marked painful area as defined by sonography before each treatment	2000*0.26–0.32	2	14 days	None
Pigozzi (2000) [37]	Prospective open design	19	38 (18–69)	> 2	NR	2000*0.21	8	1 week	None
Pleiner (2004) [38]	RCT	23 (31)	54 ± 11	> 6	SWT was focused on the point of maximum pain	2000*0.28	2	14 days	None
Rompe (1995) [39]	Prospective open design	40	47	25 (12–120)	SWT was administered once the calcium deposit is situated in the center of the C-arm.	1500*0.28	1	NA	None, yes
Rompe (2001) [40]	prospective quasi-randomized	50	49.6 ± 7.5	52.6 ± 54.4	SWT was administered once the calcium deposit is situated in the center of the C-arm	3000*0.6	1	NA	Active exercise for 4 to 6 weeks, yes
Sabeti-Aschraf (2005) [41]	RCT	25 each	52.68 ± 8.19	> 6	In group 1, the angle and distance between the SWT and shoulder were adjusted until the patient reported to the point of maximum tenderness. In group 2, the Lithotrack device was used to locate the calcium deposit in the center of a crosshairs by fluoroscopy in 2 planes. The computer calculated the angle and distance to provide maximum precision.	1000*0.08	3	1 week	None
Tornese (2011) [42]	RCT	35	52.6	NR	Group A (Neutral position): the subject lay supine with shoulder in neutral rotation, the arm placed alongside the trunk and the hand resting on the abdomen Group B (Hyperextended internal rotation): the subject lay supine with shoulder in hyperextension and internal rotation with the hand placed under the buttock and the palm facing down	1800*0.22	3	1 week	None

*NSAIDs* Nonsteroidal Anti-Inflammatory Drugs, *NA* not applicable, *NR* not reported

Plain radiographs obtained at different time points post SWT was the method used to assess imaging outcomes in 21 studies, while two studies used ultrasonography. Four studies reported the change in calcium deposit diameter (mm), 16 studies reported the number of participants demonstrating total calcification resorption and three reported on reduction or fragmentation of the calcium deposit (Table 2).

**Table 2:** Imaging outcome measures for rotator cuff calcifying tendinitis

Author (year)	SWT type	Comparator	Imaging outcome	Follow-up			
				Period	Baseline - F/U Mean ± SD	***P*** value	
						Within group	Between group
Albert (2007) [20]	High SWT	Low SWT	The radiological aspects of calcifications (i.e. type, size and location) were determined through lateral and anteroposterior shoulder views in neutral, external and internal rotation. Changes were graded as no resorption, partial resorption and total or subtotal resorption (over 80% reduction of calcified surface on anteroposterior view)	3 months	High: 6(15%) had total or subtotal resorption. 3(7.5%) had partial resorption Low: 2(5%) had total or subtotal resorption. 5(12.5%) had partial resorption	NR	NR
Cacchio (2006) [21]	R-SWT	Placebo	The radiological aspects of calcifications (i.e. type, size and location) were determined through lateral and anteroposterior shoulder views in 45 degrees of external and internal rotation were acquired. Type of calcification was evaluated according to the Gartner and Simons classification. A caliper that evaluated calcification length (in millimeters) was used for size measurement.	1 week	39(86.6%) had total resorption, 6(13.4%) had partial resorption, while the control group, no complete disappearance of calcifications was observed. The mean Calcium deposits diameter (mm) pre-SWT was 21.3 ± 7.5, post-SWT was 0.85 ± 1.2. In contrast, pre-sham was 19.7 ± 8.3, post-sham was 18.85.8 ± 6.4.	< 0.00	< 0.00
Charrin (2001) [22]	F-SWT	None	Calcific deposit appearance was assessed on a plain radiograph in neutral rotation	3,6,12,24 weeks	After 12 weeks, 2/30 had total resorption. 5 deposits had partial resorption. After 24 weeks, 5/29 had total resorption and 2 deposits had partial resorption	NR	NA
Cosentino (2003) [23]	F-SWT	Placebo	Variations in the dimension of the calcification were evaluated by anteroposterior views. Modification of the calcification (a reduction of size of > 2 mm) was indicated as disintegration; the total disappearance was indicated as dissolution.	1 month	11(31%) had total resorption, 14(40%) had partial resorption. Calcification remained unchanged in the control group	< 0.001	NR
Daecke (2002) [24]	F-SWT	1 vs 2 sessions	Anteroposterior radiograph in internal and external rotation was obtained to show obvious changes in the shape and structure (disintegration) or complete resorption of the calcification	3,6 months, 4 years	30% in group A and 52% in group B had partial or total resorption after 3 months, 47 and 77% after 6 months and 93% for both groups at 4 years	NR	< 0.046 at 6 months
DeBoer (2017) [25]	R-SWT	Ultrasound Needling (UN)	Scoring of calcification deposits was assessed through the Gartner Classification of Calcific Tendinitis.	6 weeks	1/14 (7%) in the R-SWT group had total resorption vs 5/11 (45.5%) in UN group	NR	0.029
DelCastillo-Gonzalez (2016) [26]	F-SWT	Ultrasound-guided percutaneous lavage (UGPL)	Calcification size measurement was assessed by ultrasound imaging.	3,6,12 months	55.6% had total resorption by 12 months in the SWT group vs 86.78% in the UGPL group. The mean Calcium deposits diameter (mm) pre-SWT was 10.53 ± 5.29, post-SWT was 4.67 ± 6.08 after 12 months. In contrast, pre-UGPL was 12.075 ± 4.85, post-UGPL was 1.56 ± 2.79.	< 0.01	< 0.01
Farr (2011) [27]	F-SWT	Low SWT	Radiological difference of the calcific deposit was rated as improvement, unchanged or worsening	6,12 weeks	58% improved in group A compared to 69% in group B after 12 weeks. 5 in group A, and 4 in group B had total resorption	NR	NR
Gerdesmeyer (2003) [28]	F-SWT	Low SWT and placebo	The radiological aspects of calcifications (i.e. type, size and location) were determined through anteroposterior shoulder views in 45 degrees of external and internal rotation.	3,6,12 months	High: 60% had total resorption within 6 months and 86% after 12 months. Low: 21% had total resorption within 6 months and 37% after 12 months. Placebo: 11% had total resorption within 6 months and 25% after 12 months. Calcific deposit size (mm2) mean change from baseline after 12 months was − 162.2 (95%CI − 204 to −120) in the High-SWT group, − 91.5 (95%CI − 148 to − 35.1) low-SWT group and − 46.8 (95%CI −74.3 to −19.3) in the placebo group	NR	group 1 vs 3 *P* < 0.01, group 2 vs 3 P = 0.1, group 1 vs 2 *p* = 0.04
Hsu (2008) [29]	F-SWT	Placebo	An anteroposterior radiograph with the arm in neutral rotation was obtained. The calcific deposits were categorized according to morphology and size (the longest length of the calcium deposit). Scoring of calcification deposits was assessed through the Gartner Classification of Calcific Tendinitis	6 weeks, 3,6,12 months	7(21.2%) had total resorption, 11(36.6%) had partial resorption. In the control group, none had total resorption and 2(15.3%) had partial resorption. The mean Calcium deposits diameter (mm) pre-SWT was 11.9 ± 5.4 (3.4–23.5), post-SWT was 5.5 ± 6.3 (0–18.7). In contrast, pre-sham was 10.5 ± 6.4 (2.5–20.4), post-sham was 9.8 ± 5.9 (2.3–21).	< 0.01	< 0.01
Jakobeit (2002) [30]	F-SWT	None	Diagnostic ultrasonography and radiography were used to classify the calcareous deposits in 5 categories according to their morphological appearance and size.	4 weeks	57/80 (71.25%) had total resorption. 16/80 (20%) had partial resorption	NR	NA
Kim (2014) [31]	F-SWT	Ultrasound Needling	Radiographic evaluations were performed by standard shoulder anteroposterior radiographs in neutral, internal, and external rotation together with axillary and supraspinatus outlet views to determine the size, morphology, and location of the calcific deposits. Resorption of the calcific deposit was graded as none, partial, or complete.	6 weeks, 3,6,12 months	The mean Calcium deposits diameter (mm) pre-SWT was 11 ± 1 (4.9–19.3), post-SWT was 5.6 ± 0.8. In contrast, pre-US needling was 14.8 ± 1.7 (6.6–31), post-US needling was 0.45 ± 0.3. In the SWT group, 42.6% had total resorption, 16.7% had partial resorption. In the US needling group 72.2% had total resorption and 11.1% had partial resorption.	< 0.05	=0.001
Kransy (2005) [32]	F-SWT	Ultrasound Needling combined with SWT	Anteroposterior radiographs were taken in internal and external rotation together with axial and supraspinatus-outlet views to determine the size, morphology and location of the calcific deposits.	4.1 ± 0.5	In the SWT only group, 13(32.5%) had total resorption, 14 (35%) had partial resorption. In the US needling combined with SWT group 24(60%) had total resorption and 10 (25%) had partial resorption.	NS	=0.024
Loew (1999) [33]	F-SWT	Low SWT and control	Radiographs included an anteroposterior view in internal and external rotation and a supraspinatus outlet view. Effective treatment was recorded when the calcification had completely disappeared or showed obvious resorption with inhomogeneity and reduction in size	3,6 months	There was total resorption in 4/20 in group 1, 11/20 in group 2, 12/20 in group 3, in contrast to 2/20 in the control group	Group 1 = 0.37 Group 2, 3 < 0.01	NR
Lowe (1995) [34]	F-SWT	None	Radiological assessment of the calcification was made in three different planes.	6,12 weeks	After 12 weeks, changes were seen in 12 patients; 7 showed a total resorption of the calcium deposits	NR	NA
Moretti (2005) [35]	F-SWT	None	Radiographs in anteroposterior of shoulder, acromial outlet view and sonography were evaluated to study the type of calcium deposit according to DePalma criteria	1,6 months	29/54 (54%) had total resorption and 19/54 (35) had partial resorption after 1 month. These findings appeared unvaried at 6 months follow-up	NR	NA
Pan (2003) [36]	F-SWT	TENS	High-resolution ultrasonography (HRUS) was used for imaging measurements. The morphology of calcific plaque of the shoulder on HRUS was classified into 4 types: (1) arc-shaped (echogenic arc with clear shadowing), (2) fragmented (at least 2 separated echogenic plaques with or without shadowing) or punctuated (tiny calcific spots without shadowing), (3) nodular (echogenic nodule without shadowing), and (4) cystic types (bold echogenic wall with echo-free content)	2,4,12 weeks	The mean of difference in Calcium deposits diameter (mm) in the SWT group was 4.39 ± 3.76 after 12 weeks. In contrast, the TENS group was 1.65 ± 2.83. 16/33(48.5%) changed in the type of calcification In the SWT group while 3/29 (10.3) in the TENS group.	< 0.01	0.002
Pigozzi (2000) [37]	F-SWT	None	Radiological assessment of anteroposterior, internal and external rotation and trans-glenoid projection was performed	1 month	7/19 (37%) had reduction or fragmentation of the calcium deposit	NR	NA
Pleiner (2004) [38]	F-SWT	Placebo	Anteriorposterior, axial and outlet-view images were used. Changes calcifications were assessed using the Gartner scale in which a score of 1: indicates no change or a worsening, a score of 2: a decrease of at least 50% in the area and density of the calcification, and a score of 3: complete remission of the calcification	3,7 months	6/31 (19.4%) had total resorption and 6/31 (19.4%) had partial resorption in the SWT group, in contrast to 2/26 (7.7%) had total resorption and 2/26 (7.7%) had partial resorption in the control group after 7 months	NR	=0.07
Rompe (1995) [39]	F-SWT	None	On radiographs, any sign of disintegration was rated as success	6,24 weeks	4/40 (10%) had total resorption and 17/40 (42.5%) had partial resorption after 6 weeks. After 24 weeks, 6/40 (15%) had total resorption and 19/40 (47.5%) had partial resorption	NR	NA
Rompe (2001) [40]	F-SWT	Surgical extirpation	On the anteroposterior radiological views, resorption was graded as none, partial, or complete.	12 months	47% had total resorption and 33% had partial resorption in the SWT group. In the surgical group, 85% had total resorption and 15% had partial resorption. There was no significant difference regarding the radio-morphologic features	NR	< 0.01
Sabeti-Aschraf (2005) [41]	F-SWT	Navigation vs feedback	No change in the radiographs was graded as 4, a 3 indicated slight alteration of the calcium deposit, reduction in deposit size and radiographic density was graded as 2, and a 1 was given if the calcium deposit was no longer evident	3 months	6/25 (24%) had total resorption and 7/25 (28%) had extensive resorption in the navigation group, in contrast to 1/25 (4%) had total resorption and 5/25 (20%) had partial resorption in the feedback group	NR	0.041
Tornese (2011) [42]	F-SWT	Neutral vs hyperextended internal rotation arm position	Changes between pre- and post-treatment radiographs were graded as no resorption, partial resorption and total or subtotal resorption (> 80% reduction in calcified surface on anteroposterior view)	3 months	12/18 (66.7%) had total resorption in the hyperextended internal rotation group, in contrast to 6/17 (35.3%) in the neutral position group	NR	< 0.05

*F-SWT* focused SWT, *R-SWT* radial SWT, *NR* not reported, *NS* not significant, *NA* not applicable

**Fig. 3:**
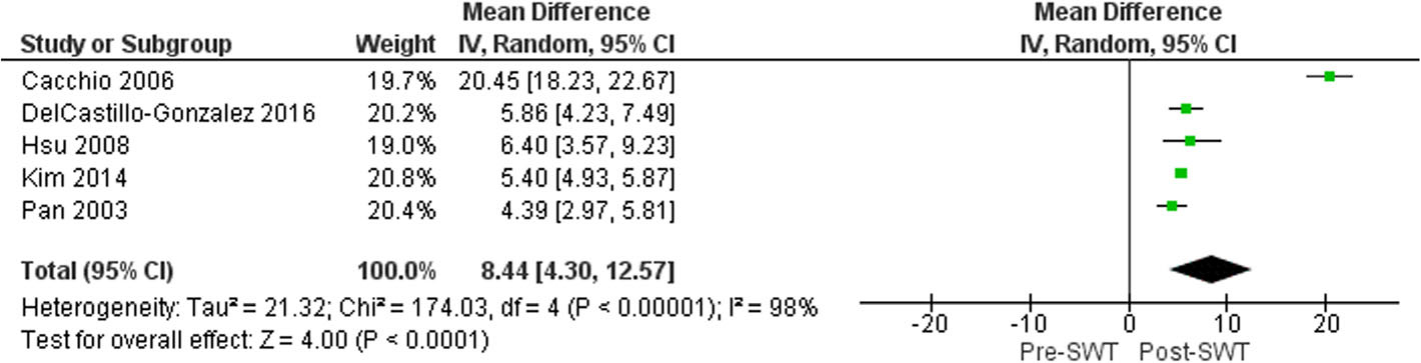
Forest plot of effect of SWT on Calcium deposit diameter (mm) in rotator cuff calcific tendinitis.

The size of calcium deposit was shown to reduce following SWT application (MD 8.44 mm (95%CI 4.30, 12.57), *p* < 0.001; Fig. 3) within a period of 1 week [21], 3 months [36] and 12 months [26, 29, 31]. Baseline calcium deposit size was the only covariate to explain the variance related to the effect of SWT (Coeff. 1.38 mm (95%CI 0.98, 1.77) I^2^ = 43.55%, Adj. R^2^ = 99.18%, *p* = 0.002). No variables related to SWT treatment parameters were significant covariates.

**Fig. 4:**
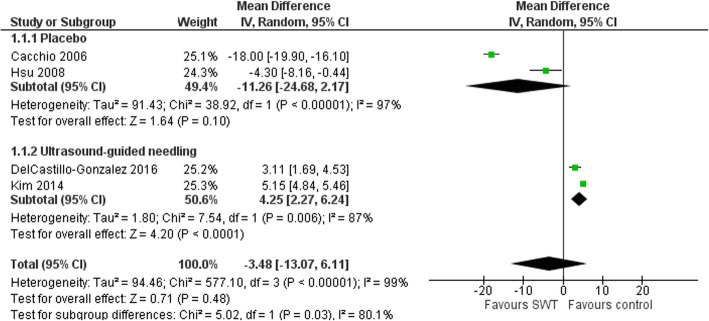
Forest plot of effect of SWT vs control on Calcium deposit diameter (mm) in rotator cuff calcific tendinitis

**Fig. 5:**
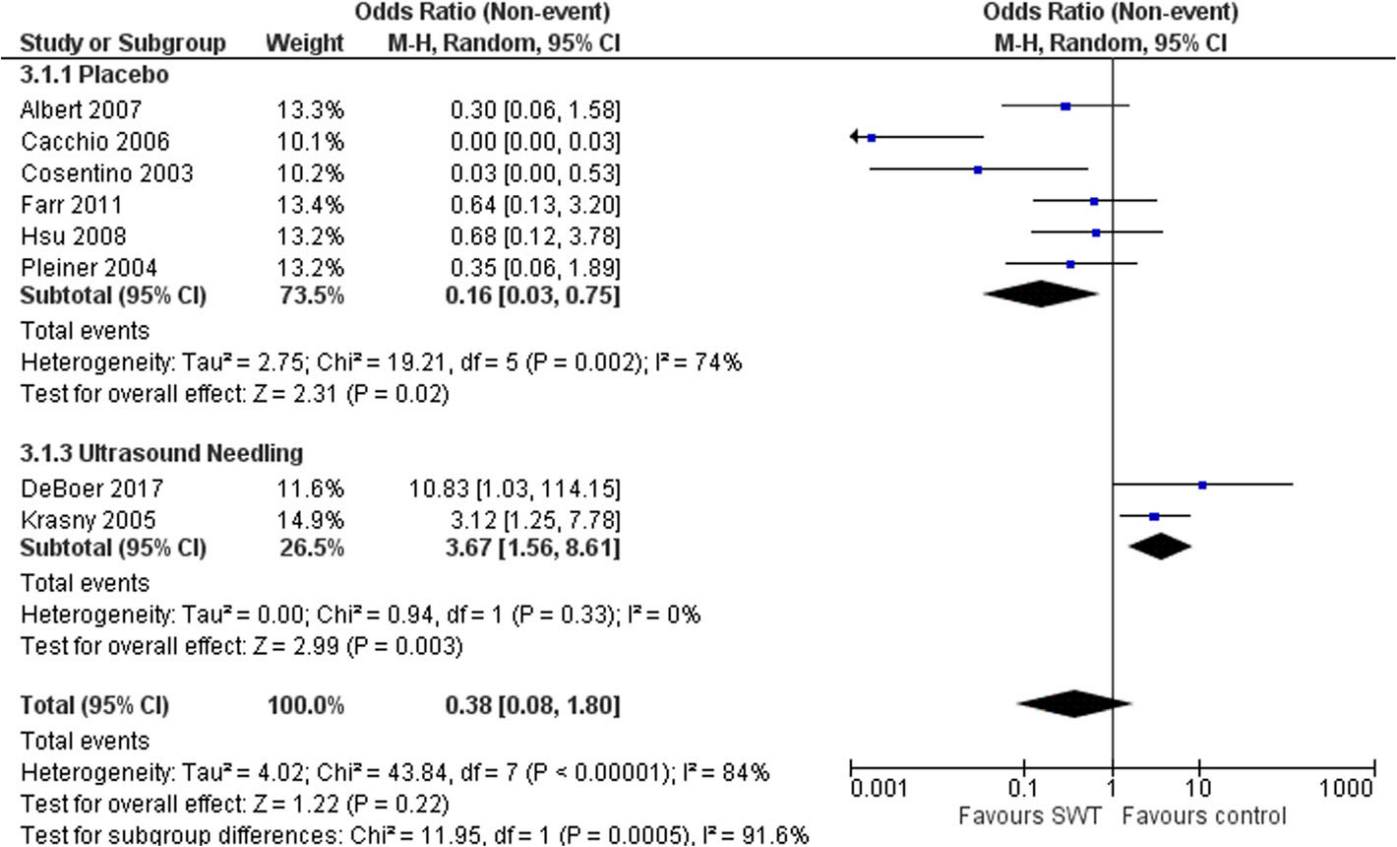
Forest plot of effect of SWT vs control on total calcification resorption in rotator cuff calcific tendinitis

The reduction in calcium deposit size favored SWT compared to placebo but did not reach statistical significance (MD − 11.26 mm (95%CI -24.68, 2.17), *P* = 0.1) [21, 29]. However, the effect of SWT on calcium deposit size was less compared to ultrasound-guided needling (MD 4.25 (95%CI 2.27, 6.24), *p* = 0.006), (Fig. 4) [26, 31]. Total calcium resorption was reported in 222/559 (35%) shoulders. Total calcium resorption was greater in the SWT group compared to placebo (OR 6.38 (95%CI 1.33, 30.70, *p* = 0.02), but not compared to ultrasound guided-needling (OR 0.27 (95%CI 0.12, 0.64), *p* = 0.003), (Fig. 5).

### Plantar fasciitis and heel spurs

A total of 13 studies (7 RCTs) published between 2008 and 2018 evaluated imaging changes in plantar fasciitis following SWT. The total number of included participants was 359 (365 heels) diagnosed with symptomatic heel pain in addition to imaging features of plantar fascia thickening (> 4 mm) as the main inclusion criteria. The main exclusion criteria were the presence of systemic disease such as rheumatoid arthritis, diabetes mellitus, vascular abnormalities or neurological impairments.

Eight studies utilized focused-SWT, radial-SWT was used in three studies and one study employed a combined focused and radial SWT type stimulus. The type of SWT was not reported in one study [43]. The mean SWT shocks per treatment session was 2426.92 ± 965.36 (1000–4000) with a mean EFD of 0.22 ± 0.14 (0.03–0.42) mJ/mm^2^. The mean number of treatment sessions was 3.04 ± 1.67 (1–8) sessions with a mean of 24.79 ± 43.56 (1–140) days between sessions. Only two studies used anesthesia before the application of SWT and four studies employed sonographic guidance to localize the area of application (Table 3).

**Table 3:** Characteristics of studies and intervention details for plantar fasciitis and heel spurs

Author	Study design	Condition	N	Mean age ±SD or (range)	Mean symptoms duration ± SD or (range), months	Area of SWT application	Dosage in impulses*EFD (mJ/mm^**2**^)/bar	No. of sessions	Interval between sessions	Co- intervention, anesthesia
Bicer (2018) [44]	Prospective open design	Plantar fasciitis	30	45.23 ± 8.57	> 6 weeks	NR	2500*2–3 bars	3	1 week	None
Chew (2013) [45]	RCT	Plantar fasciitis	19	median and interquartile range: 45 (37–53)	median and interquartile range: 18 (7–24)	SWT delivered under ultrasound guidance to the painful and thickened region of the plantar fascia at the medial calcaneal tubercle. The patient was positioned prone with the feet over the end of the table	2000*0.42	2	1 week	Home exercise program, none
Daniel-Lucian (2013) [43]	Prospective open design	Plantar fasciitis	17	NR	> 3	SWT was applied over the calcaneal plantar side, aponeurosis insertion and plantar aponeurosis	4000 EFD is NR	8	Twice a week	None
Gerdesmeyer (2015) [46]	prospective intra-individual controlled	Heel spur	45	53 (28.6–80.1)	> 6	SWT localization of the most painful area was achieved with biofeedback mechanism and radiologically controlled. SWT was placed at the origin of the fascia and reached the central calcaneus.	2000*0.32	2	2 weeks	None
Hammer (2005) [47]	prospective intra-individual controlled	Plantar fasciitis	22	51.6 (24–79)	8.8 (6–12)	NR	3000*0.2	3	1 week	None
Hocaoglu (2017) [48]	RCT	Plantar fasciitis	36	50.22 ± 8.29	8 (6–24)	Patients were in the prone position. SWT was applied on plantar fascia insertion of the calcaneus	2000*0.16	3	1 week	None
Lai (2018) [49]	RCT	Plantar fasciitis	47	54.53 ± 8.62	7.94 ± 2.92	NR	1500*029	2	2 weeks	None
Lee (2003) [50]	RCT	Calcaneal bone spurs	308	NR	> 6	SWT was applied from the plantar surface over a 2 cm circular area around the predetermined point of maximal tenderness at the plantar anthesis	1500*0.22	1 or 2	3 months	None, yes
Maki (2017) [51]	Prospective open design	Plantar fasciitis	23	55.3 (16–81)	26.9 (4–300)	SWT was applied at the plantar fascia attachment under ultrasonic guidance from the medial calcaneus. A second treatment was performed if symptoms persisted at 3 months	3800*0.36	1 or 2	3 months	None
Moretti (2006) [52]	Prospective open design	Plantar fasciitis	54	35.2 (30–42)	> 6	SWT was applied at the medial tubercle of the calcaneus, at the proximal insertion of the plantar fascia or the calcaneal spur, around the point of medial tenderness	2000*0.04	4	1 week	None
Saber (2012) [53]	RCT	Plantar fasciitis	30	34.27 ± 7.19	> 6	SWT was applied in prone position over the area of maximal tenderness and finding by ultrasonography	1000–1500*0.28	2	2 weeks	None, yes
Sorrentino (2008) [54]	RCT	Plantar fasciitis	30	Total sample = 34 women (56 ± 2.4) and 26 men (52 ± 3.7)	4	SWT was applied under ultrasonography guidance to locate the calcaneal insertion of the plantar fascia	2000*0.03	4	1 week	None
Ulusoy (2017) [55]	RCT	Plantar fasciitis	19	54.45 ± 6.9	27 ± 29.79	SWT was applied in the prone position into the areas of the painful heel, insertion of plantar fascia on the medial calcaneal area, and myofascial junction at the dorsum of the heel	2000*2.5-bar	3	1 week	Continue previous exercise program, no
Vahdatpour (2012) [56]	RCT	Plantar fasciitis	20	50.6 ± 10	> 3	SWT was targeted to the maximum local tenderness area	2000 focused+ 2000 radial*0.2	3	1 week	Exercise, NSAIDs, and heel pad for both groups, no
Yalcin (2012) [57]	Prospective open design	Heel spur	108	50.2 ± 11.3	27.4 ± 32.8	SWT was applied in prone position to the marked tender spot	2000*0.4 (4 bar)	5	1 week	None
Zhu (2005) [58]	Prospective open design	Plantar fasciitis	12 (18 ft)	49.9 (33–63)	> 6	SWT was applied to the most painful point (2–3 cm diameter) on the heel	1500*18kv	1	NA	None, yes

*NSAIDs* nonsteroidal anti-inflammatory drugs, *NA* not applicable, *NR* not reported

Ultrasonography was the most common method for evaluating the imaging outcomes as reported in nine studies, followed by MRI in four studies. The change in plantar fascia thickness (PFT) was evaluated in eight studies as the most frequently reported imaging outcome (Table 4).

**Table 4:** Imaging outcome measures for plantar fasciitis and heel spurs

Author (year)	SWT type	Comparator	Imaging outcome	Follow-up			
				Period	Baseline - F/U Mean ± SD	***P*** value	
						Within group	Between group
Bicer (2018) [44]	R-SWT	None	MRI was used to assess changes in the soft tissue and BME, plantar fascia thickness (PFT) and the presence of heel spurs. MRIs were scored semi-quantitatively. PFT was measured 1 cm from the insertion and thickness > 3 mm was considered abnormal	3 months	12/23 (52.1%) showed improvement in PFT. 21/30 (70%) and 10/19(52.6%) had improvement in soft tissue and BME respectively. No significant change in heel spur	< 0.05	NA
Chew (2013) [45]	F-SWT	Autologous Conditioned Plasma (ACP) and conventional	Ultrasonography of plantar fascia (PF) was performed to manually measure the point of maximal proximal PFT at the medial calcaneal tubercle insertion site.	1,3,6 months	The median PFT improvement in the ACP group at the 6-month follow-up was 1.3 mm compared with the SWT and conventional treatment groups, which both showed improvements of 0.6 mm.	NR	SWT vs conventional treatment =0.934 ACP vs SWT = 0.027
Daniel-Lucian (2013) [43]	NR	None	Ultrasonography was used to measure PFT	3 months	The mean PFT decreased from 5.84 to 5.21 in the females, and from 5.87 to 5.14 in the male subjects	NR	NR
Gerdesmeyer (2015) [46]	F-SWT	None	Measurements of bone mass density (BMD) and bone mass concentration (BMC) were performed with a Lunar DEXA. The square-shaped analysis field (Region of Interest, ROI) was placed in the cancellous part of the calcanei and BMD and BMC were measured.	6,12 weeks	The mean BMD (g/cm2) values changed from 0.5 ± 0.1 to 0.557 ± 0.1 in the SWT group and from 0.54 ± 0.1 to 0.52 ± 0.09 in the control group after 12 weeks. The mean BMC (g) values changed from 2.03 ± 0.38 to 2.22 ± 0.38 in the SWT group and from 2.16 ± 0.4 to 2.08 ± 0.36 in the control group after 12 weeks	0.001	< 0.01
Hammer (2005) [47]	F-SWT	None	The PFT was measured about 2 cm distal of the medial calcaneal tuberosity using ultrasonography	6,12,24 weeks	The mean PFT in 16 subjects changed from 5.2 ± 1.5 to 4.4 ± 1 after 6 months. There was no significant change of PFT on the control side	< 0.05	< 0.05
Hocaoglu (2017) [48]	R-SWT	Ultrasound-guided local corticosteroid injection	PFT and its echogenicity were examined through ultrasonography. A linear probe was positioned longitudinally over the medial tubercle of the calcaneus. PFT was measured at the proximal point of insertion of the fascia into the calcaneal tubercle. A PFT of 4 mm was considered evidence of fasciitis.	1,3,6 months	PFT was found to be significantly reduced in both groups at all measurement endpoints compared with baseline with no significant differences between groups	< 0.01	> 0.05
Lai (2018) [49]	F-SWT	Corticosteroid injection	PFT was measured at the PF insertion 5 mm distal to calcaneus tuberosity using ultrasonography.	1,3 months	At 4th week, the mean PFT changed in SWT group from 0.37 ± 0.07 to 0.46 ± 0.08 cm, and in the CSI group from 0.38 ± 0.06 to 0.43 ± 0.09 cm At the 12th week, the mean PFT changed in the SWT group from 0.37 ± 0.07 to 0.38 ± 0.07 cm, and the CSI group from 0.38 ± 0.06 to 0.39 ± 0.07 cm	NR	At 4th week =0.048 At 12th week =0.326
Lee (2003) [50]	R-SWT	Placebo	Axial, lateral, and oblique radiographs of the calcaneus were performed to examine the presence of any osseous abnormalities of the calcaneus or for the presence of inferior calcaneal spurs	3,12 months	205/308 (67%) in the EWST group had an inferior calcaneal spur. In the sham treatment group, 78/127 (61%) had a spur. No patient treated with SWT had subsequent fragmentation or disappearance of the heel spur at 3 or 12 months. Similarly, no patient had evidence of reactive new bone formation in or around the spur, nor apparent elongation of the spur	NR	NR
Maki (2017) [51]	F-SWT	None	On MRI, 4 items were examined: PFT, high-signal intensity area (HSIA) inside the PF, edema around the PF, and BME of the calcaneus. For the PFT, the maximum diameter of the PF at the calcaneal attachment was measured on T1-weighted coronal images.	6 months	The mean PFT changed from 4.4 ± 1.6 to 4.6 ± 1.8 after 6 months. The numbers of feet showing HSIA inside the PF changed from 15 to 6, in edema around the PF from 16 to 2, and in BME of the calcaneus from 11 to 4.	> 0.05	NA
Moretti (2006) [52]	F-SWT	None	A lateral weight-bearing X-ray of the foot and ultrasound evaluation was performed.	45 days, 6,24 months	There was no heel spur fragmentation observed. The ultrasound evaluation at 24 months showed a complete disappearance of the inflammatory signs in 33(61%) patients.	NR	NA
Saber (2012) [53]	F-SWT	Ultrasound-guided local corticosteroid injection	PFT was measured at the thickest portion from the base of the medial calcaneal tubercle where a bright echogenic line was easily visible using ultrasonography	20 (12–24) weeks	The mean PFT in the SWT group changed from 5.93 ± 0.54 to 3.37 ± 0.42, and in the ultrasound guided injection group from 5.96 ± 0.46 to 3.54 ± 0.31	< 0.01	=0.079
Sorrentino (2008) [54]	F-SWT	Corticosteroid injection	Ultrasonography was performed in prone position with ankles dorsiflexed. The focus was adjusted to the depth of the PF. The sonographic diagnosis established based on: 1) fascial thickening > 5 mm, 2) biconvex morphology and 3) abnormal fascial echostructure, specifically hypoechogenicity, heterogeneity and ill-defined margins. PFT was measured 1 cm from the calcaneal insertion with electronic calipers	6 weeks	In the SWT group, PFT with perifascial edema was reduced to 4.6 ± 0.6 mm and up to 4 ± 0.3 mm among PFT without perifascial edema. In the corticosteroid Injection, PFT with perifascial edema was reduced to 4.3 ± 0.4 mm and up to 4.6 ± 0.4 mm among PFT without perifascial edema	NR	NR
Ulusoy (2017) [55]	R-SWT	low-level laser therapy (LLLT) and therapeutic ultrasound (US)	The maximum thickness of the proximal PF where it attaches to the calcaneus was measured using electronic calipers on fluid-sensitive MRI sequences in the sagittal and coronal planes. The intrafacial and perifacial soft tissue edema and calcaneal BME were assessed in the sagittal plane on short tau inversion recovery sequences, and the presence of the calcaneal spurs was evaluated on T1-weighted sequences	1 month	The mean PFT in the SWT group changed from 5.17 ± 0.89 to 4.31 ± 0.82, in the LLLT group from 4.33 ± 0.59 to 3.75 ± 0.69, and in the US group, from 4.76 ± 0.72 to 3.99 ± 0.62 seen on MRI coronal plane	<.01	NS
Vahdatpour (2012) [56]	F-SWT and R-SWT	Placebo	Sagittal imaging of the PF was performed with the ultrasound transducer aligned along the longitudinal axis of the aponeurosis. PFT was measured about 2 cm distal of the medial calcaneal tuberosity. Qualitative assessment was performed including echogenic appearance of plantar fascia and its fibrillary pattern	3 months	The mean PFT in the SWT group changed from 4.1 ± 1.3 to 3.6 ± 1.2, in the placebo group from 4.1 ± 0.8 to 4.5 ± 0.9	<.01	=0.02
Yalcin (2012) [57]	R-SWT	None	Lateral radiographs evaluated variations in the dimensions of calcaneal spurs. The radiographic variations included classification as reductions in the dimensions and the angle of calcaneal spurs	NR	No significant disappearance of heel spurs, but 19(17.6%) had a decrease in the angle of the spur, 23(21.3%) had a decrease in the dimensions of the spur, and 1(0.93%) had breakage of the spur	NR	NA
Zhu (2005) [58]	F-SWT	None	Prior to MRI, a vitamin E capsule was taped to the heel that could be readily seen on MRI, where the point of maximal intensity of pain was delineated with a permanent marker. MRI assessed the presence and severity of soft tissue and calcaneal marrow edema, heel spur and PFT.	24 h	16/18(89%) had subcutaneous soft tissue and perifascial edema before SWT. After SWT, all 18 showed subcutaneous soft tissue and perifascial edema. Calcaneal marrow edema was seen in 8 heels. After SWT, edema increased in 1 heel and 1 new heel edema was developed. Heel spur was seen in 9(50%) that was unchanged. 17(94%) had an abnormal PFT (> 4 mm) before SWT that remained unchanged following SWT	NR	NA

*BME* bone marrow edema, *F-SWT* focused SWT, *R-SWT* radial SWT, *NA* not applicable, *NR* not reported, *NS* not significant

**Fig. 6:**
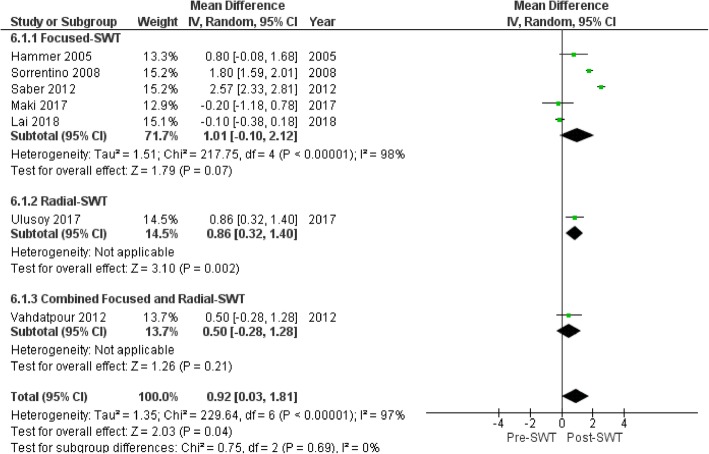
Forest plot of effect of SWT on plantar fascia thickness (mm)

**Fig. 7:**
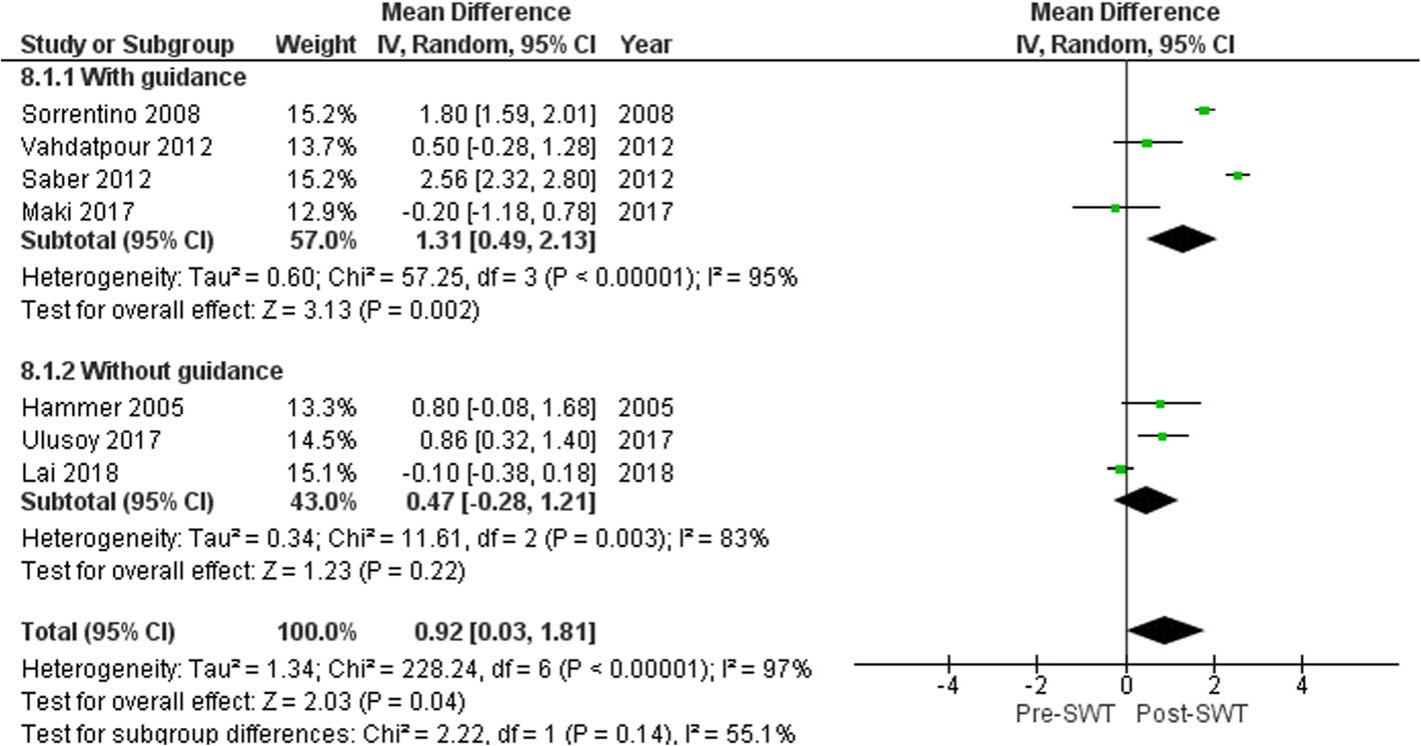
Forest plot of effect of SWT on plantar fascia thickness (mm) based on radiological guidance

**Fig. 8:**
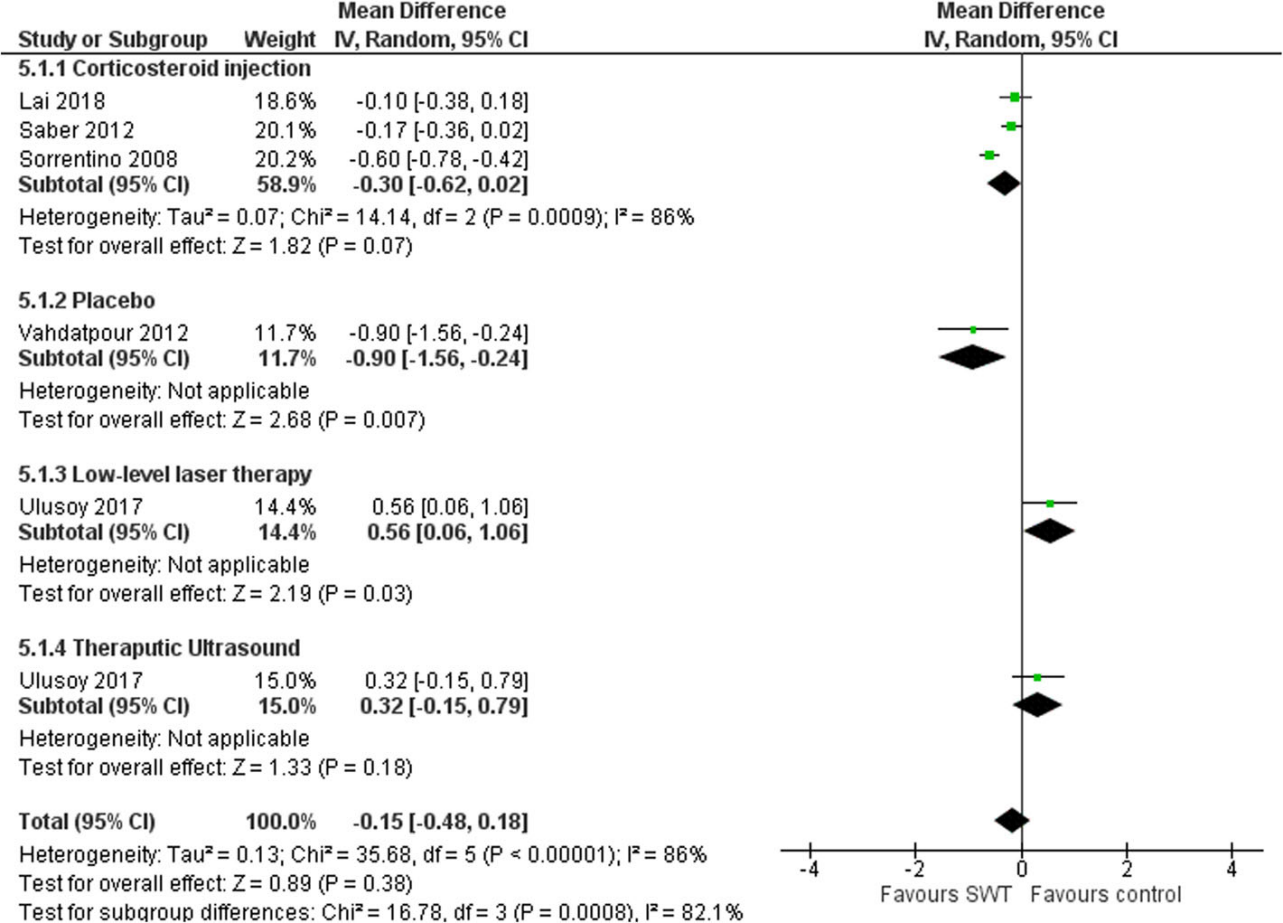
Forest plot of effect of SWT vs control on plantar fascia thickness (mm)

There was an overall reduction in PFT following SWT application (MD 0.92 mm (95%CI 0.03, 1.81), *p* = 0.04; Fig. 6) at 4 weeks [55], 6 weeks [54], 3 months [49, 53, 56] and 6 month follow-up [47, 51]. Subgroup analysis showed greater reduction in PFT using radiological guidance (MD 1.31 mm (95%CI 0.49, 2.13), *p* = 0.002) versus no guidance (MD 0.47 mm (95%CI -0.28, 1.21), *p* = 0.22) (Fig. 7). Baseline PFT was the only covariate to explain the variance related to the effect of SWT (Coeff. -1.06 mm (95%CI − 1.59 to − 0.53) I^2^ = 78%, Adj. R^2^ = 85.10%, *p* = 0.004). No variables related to SWT treatment parameters were significant covariates. The reduction of PFT favored SWT compared with corticosteroid injection (MD − 0.3 mm (95%CI -0.62-0.02), *p* = 0.07) [49, 53, 54] and over placebo control (MD − 0.9 mm (95%CI − 1.56 to − 0.24), *p* = 0.007) [56]. However, the effect of SWT on PFT was less compared to low-level laser therapy and therapeutic ultrasound (MD 0.43 mm (95%CI 0.09, 0.78), *p* = 0.01) [55] (Fig. 8).

The bone resorption effect of SWT on calcaneal spurs was evaluated in three studies (1 RCT) with a total of 461 participants. Chronic symptomatic heel pain (> 3 months) and radiological evidence of a calcaneal spur were the main inclusion criteria. Radial SWT was used in two studies and one study employed focused SWT. None of the studies utilized radiological guidance and only one study used anesthesia [50]. The range of delivered shocks was 1500–2000 shocks carried in 1–5 sessions with 7–30 days interval between each session (Table 3).

Morphological changes in calcaneal spurs were evaluated using plain radiographs in two studies, while one study [46] measured bone mineral density (BMD) and bone mineral content (BMC) using DEXA scanning. None of the studies reported calcaneal spur fragmentation or significant reduction of the spur dimensions [50, 57]. However, both BMD and BMC demonstrated statistically significant improvement following focused SWT after a 12 week follow-up period, indicating an osteogenic effect [46] (Table 4).

### Osteonecrosis of the femoral head

The effects of SWT on osteonecrosis of the femoral head (ONFH) were evaluated in 12 studies (4 RCTs) published between 2001 and 2018. These studies included 325 (404 hips) participants with stage I-III ONFH according to the Association Research Circulation Osseous (ARCO) classification as the main inclusion criterion, except for one study [59] that included ARCO stage I only. The main exclusion criteria were late ARCO stages, infection, advanced arthritis, neoplastic disease or blood coagulation disorders.

All of the included studies employed focused SWT with a mean EFD of 0.57 ± 0.06 (0.47–0.62) mJ/mm^2^ and a mean of 4867.86 ± 1469.64 (2000–6000) shocks. All of the included studies delivered the treatment over one session, except Vulpiani et al. [60] who provided treatment over four sessions and D’Agostino et al. [59] over two sessions (2–3 days interval). Anesthesia was used in eight studies and radiological guidance was implemented in nine studies to accurately target the site of lesion (Table 5).

**Table 5:** Characteristics of studies and intervention details for osteonecrosis of the femoral head (ONFH)

Author (year)	Study design	N	Mean age ±SD or (range)	Mean symptoms duration ± SD or (range), months	Area of SWT application	Dosage in impulses*EFD (mJ/mm^**2**^)/bar	No. of sessions	Interval between sessions	Co- intervention, anesthesia
Algarni (2018) [61]	Prospective open design	21 (33 hips)	37.5 ± 4.8	6 ± 3	The hip was fixed in adduction and internal rotation, ONFH was marked using fluoroscopy in 2–3 points depending on the size of the lesion	3000–4500 (1500 pulses for each 2–3 point)* 26 kV	1	NA	None, yes
Chen (2009) [62]	Prospective comparative design	17	42.9 ± 9.3	11.3 ± 3.4	Four points with 1 cm apart within the junctional zone were chosen with a metallic pin under C-arm control, and the corresponding locations were marked on the skin in the groin area. The depth of treatment was adjusted until the two ring markers of the device synchronized under C-arm imaging	1500*0.62 each of the four sites	1	NA	None, yes
D’Agostino (2014) [59]	Prospective open design	20	43.23	4.2 (4–7) weeks	NR	4000*0.5	2	2 days	None
Hsu (2010) [63]	RCT	35 (48 hips)	39.6 ± 11.9	7.2 ± 2.9	The hip joint was properly positioned by abduction and internal or external rotation. The junctional zone between avascular and normal bones of the femoral head was delineated with C-arm imaging	1500*0.62 each of the four sites	1	NA	None, yes
Ludwig (2001) [64]	Prospective open design	22	54.9 ± 12.3	NR	NR	4000*0.62	1	NA	None
Vulpiani (2012) [60]	Prospective open design	36	Stage I: 49.3 ± 11.9 Stage II: 52.7 ± 14.6 Stage III: 45.9 ± 14.1	Stage I: 4.3 ± 2.4 Stage II: 9.3 ± 4.6 Stage III: 14.7 ± 5.9	SWT was focused around (on the margins of) the necrotic bone of the femoral head under radiographic guidance	2400*5	4	2–3 days	None
Wang (2016) [65]	RCT	33 (42 hips)	41.8 ± 9.1	9.3 ± 8.4	Both legs were properly positioned. Under C-arm and MRI guidance, the junctional zone between normal bone and necrotic bone within the femoral head was delineated. Within the junctional zone, four points approximately 1 cm apart were chosen under C-arm imaging control and the corresponding locations were marked on the skin in the groin area	Group A: 2000*0.51 Group B: 4000*0.51 Group C: 6000*0.51	1	NA	None, Yes
Wang (2012) [66]	Prospective comparative design	23 (29 hips)	NR	NR	NR	6000*0.474	1	NA	None
Wang (2009) [67]	Prospective comparative design	Total 39, 15(26 hips) with SLE, 24(29 hips) controls	SLE group: 32.33 ± 8.97 Non-SLE group: 36.47 ± 8.95	SLE group: 6.88 ± 2.63 Non-SLE group: 7.1 ± 2.79	Four points with 1 cm apart within the junctional zone were chosen with a metallic pin under C-arm control, and the corresponding locations were marked on the skin in the groin area. The depth of treatment was adjusted until the two ring markers of the device synchronized under C-arm imaging	1500*0.62 each of the four sites	1	NA	None, yes
Wang (2005) [68]	RCT	23(29 hips)	39.8 ± 12.1	5.9 ± 4.5	SWT was applied in the supine position. The hip was positioned in adduction and internal rotation. In patients with a stage-II or III lesion, the junctional zone between avascular and vascular bone of the femoral head was delineated under c-arm control. Four focal points 1 cm apart, within the junctional zone were selected, and the corresponding locations on the skin in the groin area were marked. In patients with a stage-I lesion, the junctional zone was selected on the basis of findings on MRI	1500*0.62 each of the four sites	1	NA	None, Yes
Wang (2008) [69]	RCT	25 (30 hips)	38.6 ± 12.6	7.5 ± 3	The junctional zone between the avascular and normal bones of the femoral head was delineated with C-arm imaging. Four points with 1 cm apart within the junctional zone were chosen with a metallic pin under C-arm control, and the corresponding locations were marked on skin in the groin	1500*0.62 each of the four sites	1	NA	None, yes
Wang (2011) [70]	Prospective open design	35 (47 hips)	38.8 ± 11.9	7.4 ± 3	The hip joint was properly positioned by adduction and internal or external rotation. Four points with 1 cm apart within the junctional zone were chosen with a metallic pin under C-arm control, and the corresponding locations were marked on the skin in the groin area. The depth of treatment was adjusted until the two ring markers of the device synchronized under C-arm imaging	1500*0.62 each of the four sites	1	NA	None, yes

*NR* not reported, *NA* not applicable

Imaging changes of ONFH were measured utilizing MRI in addition to radiography in all included studies to evaluate the lesion size, femoral head congruency, presence of a crescent sign, BME and degenerative changes of the hip joint. The percentage of change in the osteonecrosis lesion size was reported in eight studies (Table 6).

**Table 6:** Imaging outcome measures for osteonecrosis of the femoral head (ONFH)

Author (year)	SWT type	Comparator	Imaging outcome	Follow-up			
				Period	Baseline - F/U Mean ± SD	***P*** value	
						Within group	Between group
Algarni (2018) [61]	F-SWT	None	Anteroposterior and lateral radiographs were obtained to assess the size of the lesion, the extent of subchondral bone collapse, and the presence of degenerative changes in the hip joint. MRI was performed to evaluate BME, the size of the lesion, femoral head congruency, the presence of a crescent sign, and degenerative changes in the hip joint	6,12,24 months	The mean size of the lesion (%) over the femoral head pre-SWT was 59 ± 32 and post-SWT was 28 ± 16. Significant reduction in BME was noted following SWT	*p* = 0.24	NA
Chen (2009) [62]	F-SWT	Total hip arthroplasty	MRI were assessed for the congruency of the femoral head, crescent sign, the size and stage of the lesion and bone marrow edema	41 ± 7.4 month	The mean size of the lesion (%) over the femoral head pre-SWT was 23.1 ± 22.2 and post-SWT was 22 ± 23.3. Significant reduction in BME was noted after treatment	lesion size =0.466 BME =0.031	NA
D’Agostino (2014) [59]	F-SWT	None	MRI examination was performed and calculated the edema area using the Sectra PACS software	2,3,6 months	Pre-treatment, the mean edema area (mm2) was 981.9 ± 453.2. After 2 months was 469.5 ± 306.8. At 6 months, the mean edema area had reduced to 107.8 ± 248.1.	< 0.01	NA
Hsu (2010) [63]	F-SWT	Cocktail therapy (SWT, hyperbaric oxygen therapy (HBO), alendronate)	Radiographs were used to assess the size and location of the lesion, congruency of the femoral head, the presence of a crescent sign and degenerative changes of the hip joint. MRI was used to evaluate the changes in the size of the lesion, the congruency of the articular surface of the femoral head and BME	6,12 months	The mean size of the lesion (%) over the total femoral head surface was 28.9 ± 14.9 and 27.4 ± 18 before treatment, and 27.6 ± 14.5 and 26.2 ± 18.5 after treatment for the Cocktail therapy group and SWT alone group, respectively	=0.373 for the lesion size, =0.033 for the BME	=0.344
Ludwig (2001) [64]	F-SWT	None	Radiography and MRI were used to classify the lesions on the ARCO scale	1 year	complete healing in 4 patients, a significant decrease in the size of the area of poor circulation in 6 patients, and unchanged in 4 patients	NR	NA
Vulpiani (2012) [60]	F-SWT	None	Antero-posterior and lateral radiographs were used to evaluate the size of the lesion, the extent of collapse of subchondral bone and degenerative changes of the hip joint. MRI was used to measure the size of the lesion, assess the congruency of the femoral head, the presence of a crescent sign and/or degenerative changes, with the aim to stage the lesion according to ARCO scale	3,6,12,24 months	At all follow-up time points, the lesions show no or only minimal changes. Neither regression nor progression of lesions that had been graded before treatment as ARCO stage I and II were seen.	NR	NA
Wang (2016) [65]	F-SWT	Low vs medium vs high dosage SWT	The necrotic areas of femoral heads on MRI were estimated on a high resolution monitor via the PACS system. The percentage of the infarcted femoral head volume (IFHV) was measured by the infarcted femoral head volume divided by total femoral head volume. BME around the necrotic regions were graded on MRI as follows: grade 0: no BME; grade 1: peri-necrotic; grade 2: edema extending to femoral head; grade 3: edema extending to femoral neck and grade 4: edema extending to intertrochanteric region	6 months	The mean size of the lesion (%) in group A pre-SWT was 35.1 ± 9.4 and post-SWT was 34.2 ± 5.9, group B pre-SWT was 36.2 ± 8.6 and post-SWT was 36.6 ± 7.7 and group C pre-SWT was 30.5 ± 13.1 and post-SWT was 30.2 ± 7.3. The IFHV of lesion (%) in group A at pre-SWT was 20.8 ± 18.7 and post-SWT was 19.3 ± 19, group B at pre-SWT was 23 ± 14.1 and post-SWT was 22.5 ± 16.4, and group C at pre-SWT was 22.3 ± 15.7 and post-SWT was 18.9 ± 12.5. The stage of the lesion showed no significant differences in all groups. However, BME on MRI was significantly reduced after SWT in group C (*P* = 0.039).	> 0.05 except for the IFHV of lesion in group C = 0.028	> 0.05
Wang (2012) [66]	F-SWT	Core decompression	MRI was used to examine the size of the lesion, congruency of the femoral head, the presence of a crescent sign, BME and degenerative changes of the hip joint. The percentage of IFHV was measured by IFHV divided by total femoral head volume.	1,2,9–8 years	The mean size of the lesion (%) over the femoral head pre-SWT was 21 ± 41 and post-SWT was 30 ± 20, 30 ± 20, 26 ± 18 at 1,2, 8–9 years respectively. In the pre-surgical group was 40 ± 23, and post-surgical was 42 ± 15, 41 ± 27, 41 ± 4 at 1,2, 8–9 years respectively.	> 0.05	< 0.05 for the size of the lesion and reduction of BME after SWT
Wang (2009) [67]	F-SWT	None	Radiographs were used to assess the size of the lesion, congruency of the femoral head, the presence of a crescent sign and degenerative changes. MRI was used to evaluate the changes in lesion size, the congruency of the articular surface and BME.	1,3,6,12,24 months	The mean size of the lesion (%) over the femoral head pre-SWT in the SLE group was 34.8 ± 21.1 and 28.7 ± 14.2 post-SWT, in the Non-SLE group, pre-SWT was 32.9 ± 22.4 and post-SWT was 26.7 ± 12.9. both groups showed significant reduction of BME following SWT	> 0.05	> 0.05 for the size of lesion and < 0.05 for reduction of BME
Wang (2005) [68]	F-SWT	core decompression and bone-grafting	Radiographs of the hip joint were used to evaluate the size of the lesion, the extent of collapse of subchondral bone, and degenerative changes of the hip joint. MRI was used to examine the size of the lesion, the congruency of the femoral head, the presence of a crescent sign, BME, and degenerative changes of the hip joint	3,6,12,24 months	The mean size of the lesion (%) over the femoral head pre-SWT was 61 ± 41 and post-SWT was 30 ± 20 at 24 months follow-up. In contrast, the pre-surgical was 40 ± 23 and post-surgical was 41 ± 27. In the SWT group, 5 lesions (3 stage I and 2 stage II) regressed and 4 (2 stage II and 2 stage III) progressed. In the surgical group, 4 lesions regressed, 15 (14 stage II and 1 stage I) progressed, and 9 were unchanged	=0.282	< 0.01
Wang (2008) [69]	F-SWT	SWT + alendronate	Radiographs of the hip joint were used to assess the size of the lesion, congruency of the femoral head, the presence of a crescent sign and degenerative changes of the hip joint. MRI was used to examine the size of the lesion, the congruency of the femoral head, the presence of a crescent sign, BME, and degenerative changes of the hip joint	3,6,12,24 months	The mean size of the lesion (%) over the femoral head pre-SWT was 27.7 ± 15.5 and post-SWT was 25.7 ± 16.2 at 6 months follow-up. In contrast, the pre-SWT+ alendronate group was 32.6 ± 19.9 and post- SWT+ alendronate was 29.32 ± 21.99. Significant reduction in BME was noted in both groups.	=0.679	0.145
Wang (2011) [70]	F-SWT	None	Radiographs in AP and lateral views were used to assess the size of the lesion, congruency of the femoral head, the presence of a crescent sign and degenerative changes. MRI was used to evaluate the size of the lesion, the collapse of femoral head and BME.	6,12 months	The mean size of the lesion (%) over the femoral head pre-SWT was 27.23 ± 18.9 and 27.04 ± 19.17 post-SWT. Significant improvement in BME was noted following SWT	> 0.05 for the size of lesion and = 0.04 for reduction of BME	NA

*BME* bone marrow edema, *F-SWT* focused SWT, *R-SWT* radial SWT, *NR* not reported, *NS* not significant, *NA* not applicable

**Fig. 9:**
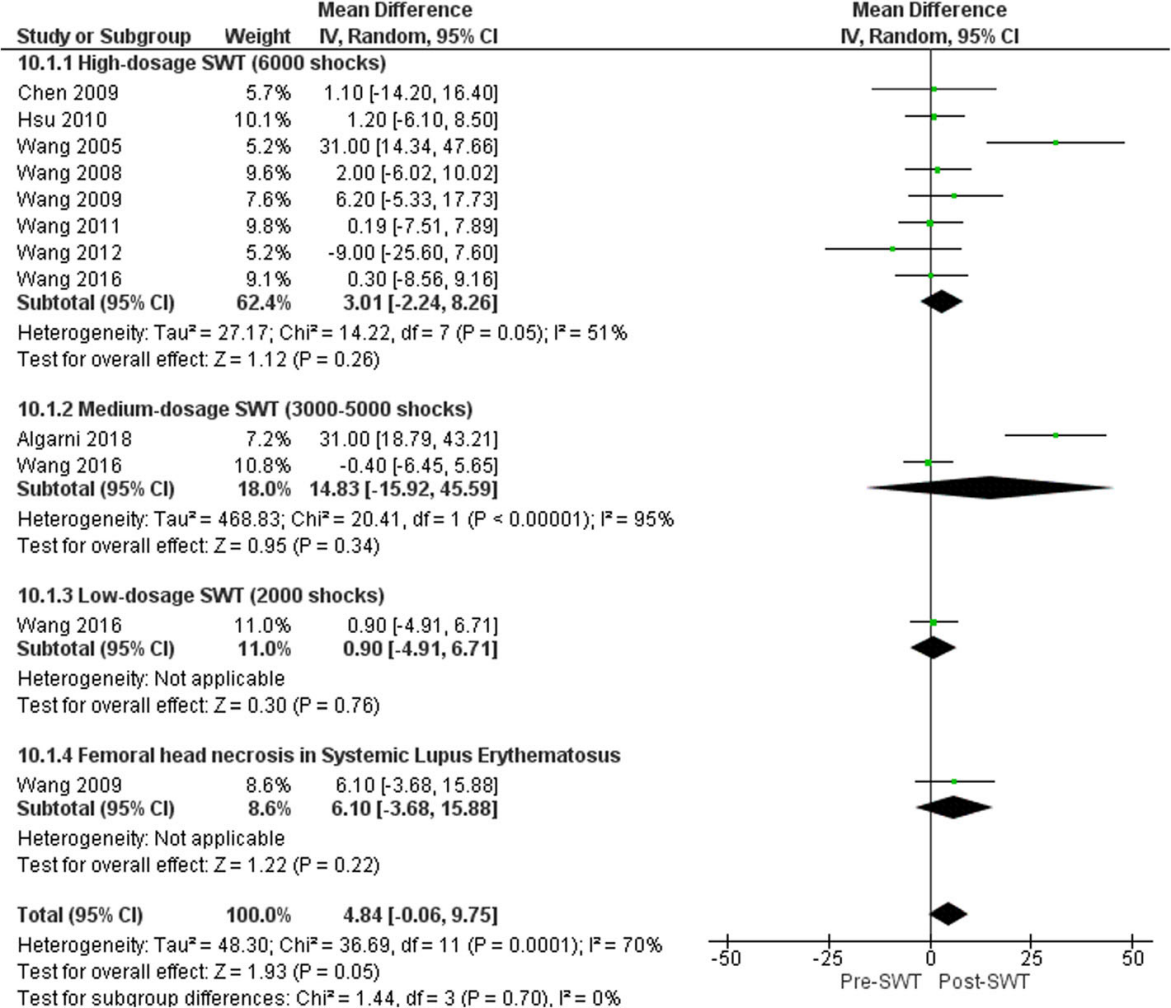
Forest plot of effect of SWT on femoral head necrosis lesion size (%)

**Fig. 10:**
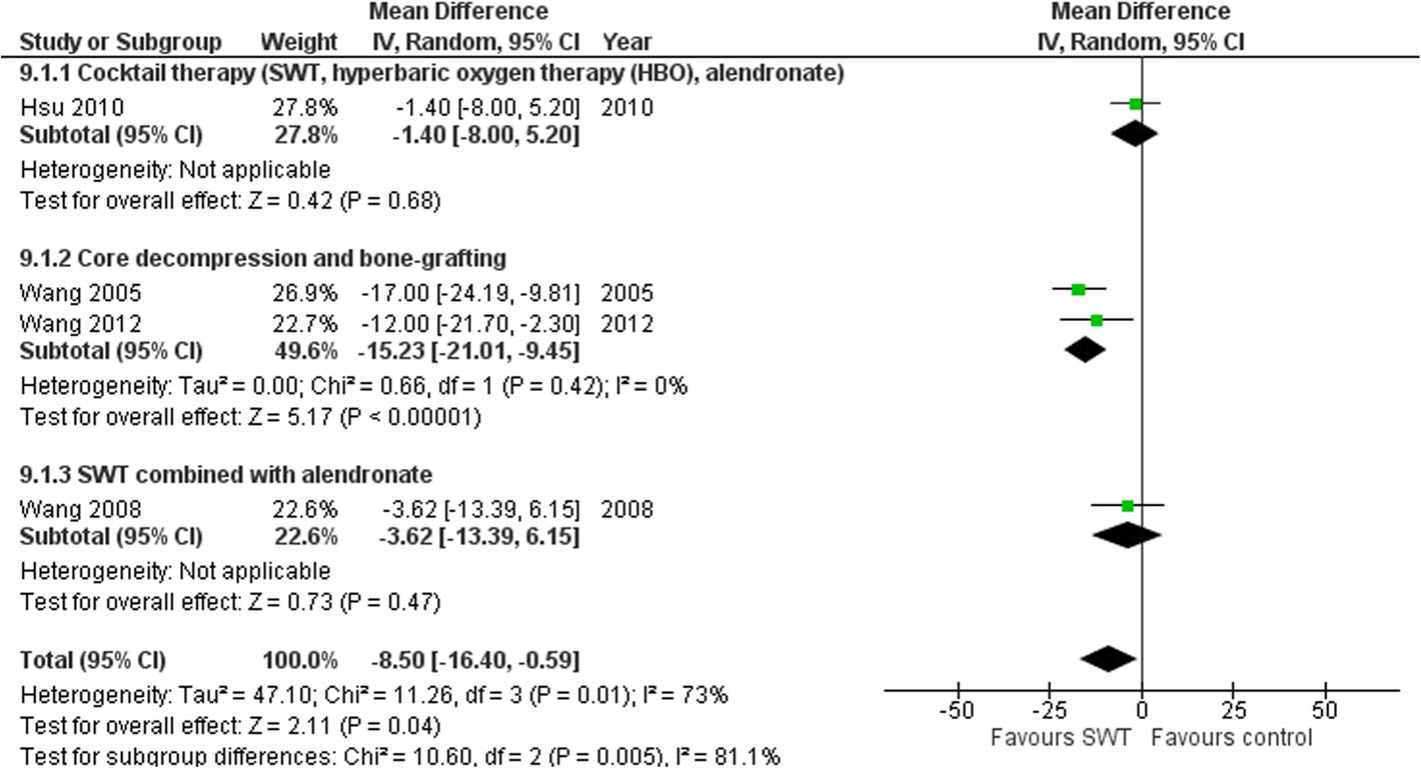
Forest plot of effect of SWT vs control on femoral head necrosis lesion size (%)

The size of the lesion (%) showed modest reduction following SWT application with marginal statistical significance (MD 4.84% (95%CI -0.06-9.75), *p* = 0.05; Fig. 9) at 6 months [65], 12 months [70], 2 years [61, 63, 66–69] and 3 years [62] follow-up. Baseline lesion size was the only covariate to explain the variance related to the effect of SWT (Coeff. 0.87% (95%CI 0.48, 1.26) I^2^ = 6.2%, Adj. R^2^ = 93.77%, *p* = 0.001). No variables related to SWT treatment parameters were significant covariates. The reduction in the lesion size generally favored SWT compared to other interventions such as core decompression [66, 68], cocktail therapy [63] and SWT combined with alendronate [69] with an overall MD -8.50% (95%CI − 16.40 to − 0.59), *p* = 0.04; Fig. 10.

### Miscellaneous conditions

The remaining musculoskeletal conditions evaluated with imaging measures following SWT were fracture non-union [71–74], lateral epicondylitis [75, 76], knee osteoarthritis related BME [77, 78], Achilles tendinopathy [79], post-traumatic myositis ossificans [80], arthroscopic rotator cuff repair [81] and Kienbock’s disease [82]. Description of these studies is provided in Additional file 3.

## Discussion

The aim of the current systematic review and meta-analysis was to evaluate changes in the morphology of musculoskeletal structures as measured by imaging following a SWT (focused and radial) intervention. Overall, there was a tendency for SWT to demonstrate morphological changes among most of the included musculoskeletal conditions based on different quantitative imaging methods. These tissue changes tended to favor SWT over placebo or other comparators except for rotator cuff calcific tendinitis that favored ultrasound guided needling over SWT. Interestingly, SWT type (radial and focused) and the therapeutic dosage parameters did not appear to have a significant influence on the evaluated imaging outcomes according to our subgroup and meta-regression analyses. Also, the utilization of imaging guidance and use of anesthesia had no clear impact on the evaluated imaging outcomes. The baseline size of the lesion was the only factor that explained the heterogeneity in our findings. However, these results should be interpreted with caution due to the relatively small overall number of studies and several potential sources of heterogeneity such as variation in study design, variation in imaging methods and measures, time period for imaging follow-up, high risk of bias and the small number of trials included in the subgroup and meta-regression analyses. In addition, SWT device related factors may contribute to the heterogeneity such as different SWT types. Further research is required to clearly determine whether there are differences in response to focused or radial SWT for different conditions.

Our meta-analysis data on the effect of SWT on rotator cuff calcific tendinitis were comparable to a recent meta-analysis [4] that reported total resorption occurred more commonly using high- versus low-energy SWT at 3 months (OR: 3.4 (95% CI 1.35,8.58); *p* = 0.009) based on 3 included studies of 163 participants. In addition to reporting the chances of total resorption versus control, the current meta-analysis has also reported within and between groups changes in the size of the calcium deposit diameter that have not been reported previously. A small number of studies (5/23) reported quantification of the change in the size of calcium deposit diameter, which limited the power of the current meta-analysis. Out of the five included studies in the meta-analysis, Cacchio et al. [21] reported the highest rate of total resorption (86.6%) and reduction in calcification deposit size MD = 20.45 mm (18.23, 22.67) at one week follow-up using a radial SWT device. The authors themselves did not expect this high rate of resorption and attributed it to the feature of radial SWT that insures the whole calcification area is included inside the wave propagation area. The only available included comparable study is by De Boer et al. [25] that also used a radial SWT device with similar treatment parameters demonstrating a total resorption of only 7% at six weeks. Our data comparison could provide an explanation related to the initial size of the calcium deposit that was exceptionally high in the Cacchio et al. [21] study (21.3 ± 7.5 mm), which was the only significant predictor in our meta-regression analysis. Larger deposits being more responsive to treatment.

According to our meta-analysis data, SWT demonstrated significant reduction in PFT and associated calcaneal BME. The reduction in PFT was greater when utilizing radiological guidance, which might be due to more consistent targeting of the SWT to the affected tissue area and avoiding surrounding areas. Although one study [55] using radial SWT showed greater reduction in PFT over focused or combined SWT, these results should be interpreted with caution based on the small number of studies included in the subgroup analysis. Despite the observed overall reduction in PFT in our review that can be correlated with the improvement in chronic plantar pain [9], it remains unclear whether the type of SWT generation device is an important factor for providing the best outcomes [2].

Our meta-analysis data revealed non-significant reduction in the lesion size with high-over medium and low-dosage SWT for ONFH. The SWT dose parameters were fairly consistent among all the included studies. This could be attributed to the same research group implementing similar treatment protocols. There are no previously published reviews or meta-analyses reporting on imaging changes following SWT among patients with ONFH to allow results comparisons. However, the observed modest reduction in the size of the lesion could be correlated with a recently published meta-analysis [8] that reported significant differences between SWT and control groups in pain rating and motor function measures.

Overall, the reported studies support changes in morphological features based on imaging findings that may reflect changes in underlying pathophysiological processes. It would appear that SWT has a clear influence on the morphology of the reported conditions, although for some conditions there is evidence to suggest that other treatments may have a greater influence on the underlying pathophysiology and associated morphological changes.

### Limitations of the study

Our aim was to include studies on all kinds of relevant musculoskeletal conditions that had been treated with any type of SWT and reporting any imaging outcomes. We conducted a comprehensive search strategy by including all possible synonyms to avoid missing any potential relevant trials, hence reducing publication bias. To date, we are not aware of any previous systematic reviews that evaluated changes in musculoskeletal conditions based on imaging outcomes following SWT interventions. Publication bias was not evaluated as there were < 10 trials for each included condition; hence, the power of the test would be very low to distinguish real asymmetry from chance.

The risk of bias scores were 60% for the RCT studies and 74% for non-RCT studies indicating relatively high risk of bias. Participant blinding, allocation concealment, study size calculation and other sources of bias (defined according to imaging assessment accuracy) were the lowest scored items. Of particular interest to this review, the accuracy of imaging measurements was questionable in some cases due to the under-reporting of details pertaining to measurement standardization. It was decided that meeting a minimum of two out of an a priori set of four criteria related to imaging accuracy would be used for judging risk of bias based on imaging accuracy. These criteria were based on providing details on the experience or specialty of the radiologist in musculoskeletal imaging, details of the imaging procedure to insure participant’s consistent position during all image acquisition, prior testing or training of the assessor to ensure reliability and reporting the score of measurements based on the average of multiple measurements.

### Future research recommendations

Current available research has provided preliminary evidence related to the capacity of SWT to influence underlying pathophysiological processes in various musculoskeletal conditions as demonstrated through changes in imaging. However, considering more standardized and reliable quantitative imaging measures as a primary outcome would be warranted in future research. This can be achieved through improving the imaging outcomes assessment methodology to ensure consistent and valid reporting based on our suggested criteria for imaging assessment accuracy. Adopting such criteria can limit the imaging assessment procedure variations as it is challenging to account for it as a covariate in the intervention effect estimate. Imaging endpoints are recommended to be specified and reported to evaluate short, medium and long term changes. Study sample sizes should be calculated based upon imaging parameters as a primary outcome. It would also be of great value if researchers could reach consensus on the optimal imaging modality and relevant imaging measures for each musculoskeletal disorder. Consistency of approach would significantly improve the quality of research.

It was surprising that our comprehensive search strategy did not identify any studies using imaging outcomes for commonly treated tendinopathies such as patellar, proximal hamstring and rotator cuff non-calcific tendinopathies and identified only a few trials for Achilles and wrist extensor tendinopathies. This is an indication of the limited use of imaging as a measure of outcome in addition to the usual clinical outcome measures for this type of condition. Whilst clinical outcomes are clearly of primary importance, imaging does provide a window to assist us in understanding the effects and potential mechanism of action of SWT. Future trials might consider making increased use of imaging outcomes in studies of this nature. This would assist in developing an improved understanding of the extent to which SWT has a therapeutic influence on pathophysiological processes in chronic musculoskeletal disorders.

## Conclusions

The current review has identified some changes in imaging parameters of musculoskeletal conditions in response to SWT. Apparently, dosage parameters of SWT had no clear influence on the imaging outcomes. Also, the utilization of radiological guidance and local anesthesia is questionable. However, the size of lesion is found to be a potential predictor for change in response to SWT. Limitations related to imaging modality selection, timing of imaging and adequate reporting of imaging procedures were factors that influenced the conclusions that could be drawn from the review.

## Supplementary information

**Additional file 1.**

**Additional file 2.**

**Additional file 3.**

## Data Availability

All data generated or analyzed during this study are presented in this published article and its additional information files. Meta-analysis data can be requested from authors.

## Abbreviations

ARCO: Association Research Circulation Osseous
BMC: Bone mineral content
BMD: Bone mineral density
BME: Bone marrow edema
CI: Confidence interval
CT: Computed tomography
DEXA: Dual-energy x-ray absorptiometry
EFD: Energy flux density
SWT: Shockwave therapy
MD: Mean difference
MINORS: The methodological index for non-randomized studies
MRI: Magnetic resonance imaging
ONFH: Osteonecrosis of the femoral head
OR: Odds ratio
PF: Plantar fascia
PFT: Plantar fascia thickness
RCT: Randomized controlled trial
SD: Standard deviation
US: Ultrasonography
