# Supplementary material for: The effects of shockwave therapy on musculoskeletal conditions based on changes in imaging: a systematic review and meta-analysis with meta-regression
Source: BMC Musculoskelet Disord. 2020 Apr 28;21:275. doi: 10.1186/s12891-020-03270-w (PMC7189454; doi:10.1186/s12891-020-03270-w)
Supplement: Supplementary file 1 — Additional file 1. [file 12891_2020_3270_MOESM1_ESM.docx]

**Ovid MEDLINE on 10/10/2018**

| **Search ID** | **Search terms** |
| --- | --- |
| 1 | extracorporeal shockwave therapy OR shockwave therapy OR shockwave OR shock wave OR shock-wave OR ESWT OR SWT |
| 2 | ultrasound imaging OR ultrasound OR sonography OR sonogram OR ultrasonography OR radiography OR MRI OR MR imaging OR magnetic resonance imaging |
| 3 | tendinopathy OR tendinosis OR tendinitis OR tenosynovitis OR calcific tendonitis OR calcific tendinitis OR calcific tendinopathy OR adhesive capsulitis OR frozen shoulder OR rotator cuff OR supraspinatus OR elbow OR common extensor OR epicondylitis OR epicondylalgia OR bursitis OR plantar fasciitis OR heel pain OR arthritis OR arthrosis OR arthralgia OR arthropathy OR osteonecrosis OR osteoarthritis OR degenerative OR fracture OR bone marrow edema OR bone marrow disease OR bone marrow lesion |
| 4 | 1 AND 2 AND 3 |
| 5 | limit 4 to (English language and humans) |
