# Supplementary figures and images for: The effects of shockwave therapy on musculoskeletal conditions based on changes in imaging: a systematic review and meta-analysis with meta-regression
Source: BMC Musculoskelet Disord. 2020 Apr 28;21:275. doi: 10.1186/s12891-020-03270-w (PMC7189454; doi:10.1186/s12891-020-03270-w)

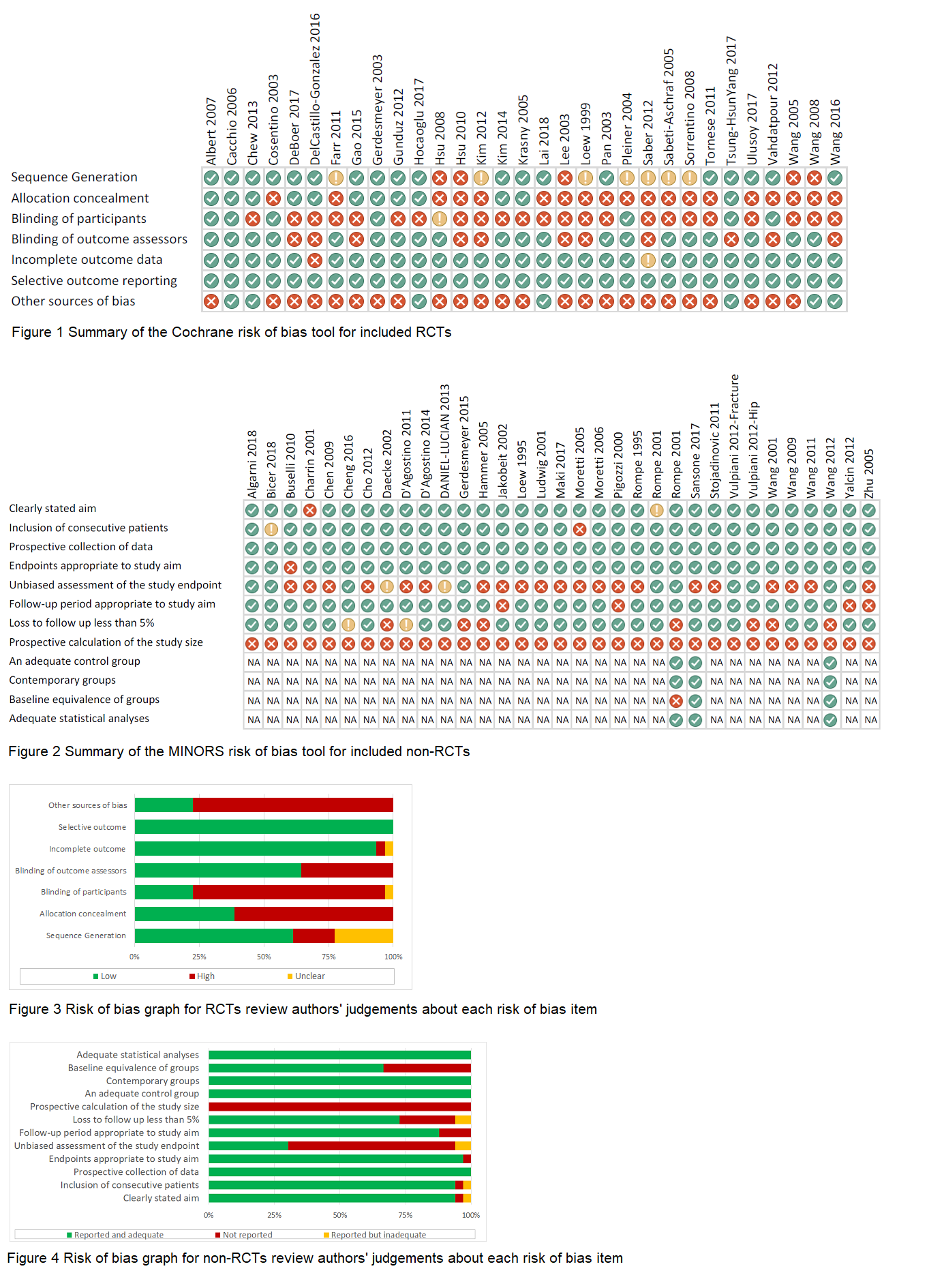

Supplement: Supplementary file 2 — Additional file 2. [file 12891_2020_3270_MOESM2_ESM.png]
