# Supplementary material for: The effects of shockwave therapy on musculoskeletal conditions based on changes in imaging: a systematic review and meta-analysis with meta-regression
Source: BMC Musculoskelet Disord. 2020 Apr 28;21:275. doi: 10.1186/s12891-020-03270-w (PMC7189454; doi:10.1186/s12891-020-03270-w)
Supplement: Supplementary file 3 — Additional file 3. [file 12891_2020_3270_MOESM3_ESM.docx]

## **Fracture non-union**

Four studies were identified evaluating the effect of ESWT on fracture non-union published between 2001 and 2012 [1-4]. All of the included studies used a prospective cohort design and enrolled a total of 607 participants. Criteria for inclusion were non-union defined as a fracture that failed to demonstrate radiological evidence of cortical bridging following six to nine months of operative or non-operative intervention. Exclusion criteria were pathological fractures, bone gap more than 5 mm, proximity to epiphyseal growth plate, local neoplasm or infection.

Focused-ESWT was used for all studies with an average EFD of 0.52±0.09 (0.25-0.84) mj/mm^2^ and an average of 3312.5±554.34 (1000-12000) shocks. Anesthesia was used in all studies and radiological guidance was employed in three studies except for Stojadinovic et al [2] ([Table 1](#_Table_7_Characteristics)).

Evaluation of fracture healing was performed via radiography based on re-establishment of cortical bridging of a minimum of three of four cortices or if no gap could be detected. The mean complete healing rate was 58.54±15.61% (40-80.8%) ([Table 2](#_Table_8_Imaging)).

## **Miscellaneous conditions**

The remaining musculoskeletal conditions evaluated with imaging outcomes following ESWT were lateral epicondylitis [5, 6], knee osteoarthritis related bone marrow edema (BME) [7, 8], Achilles tendinopathy [9], post-traumatic myositis ossificans (MO) [10], arthroscopic rotator cuff repair [11] and Kienbock's disease [12].

Two studies evaluating imaging changes in lateral epicondylitis following ESWT had a total of 50 participants with persistent lateral elbow pain for a minimum of three months. NS Cho, YH Park, JH Hwang, YC Yoon, MJ Park, JC Yoo and WH Park [5] employed focused-ESWT using four sessions weekly of 2000 shocks with 0.06-0.12 mj/mm^2^ intensity, while R Gunduz, FU Malas, P Borman, S Kocaoglu and L Ozcakar [6] employed radial-ESWT using ten daily sessions of 500 shocks with 1.4 bar pressure. The common extensor tendon thickness (mm) changed from 43(26-52) to 39.5(22-52) following ESWT (p>0.05) at six months follow-up [6]. The echotexture of the common extensor tendon showed loss or decrement of tendon thickening or recovery of normal fibrillary tendon architecture in 73% of participants with tendinosis only, 71% with tendinosis and tear and 33% with tendinosis and calcification [5].

A total of 75 participants with bone marrow edema of the knee based on MRI evidence of a bone high-intensity signal area on T2-weighted sequences were included to evaluate the effect of ESWT. Both studies employed focused-ESWT with F Gao, W Sun, Z Li, W Guo, N Kush and K Ozaki [7] using two weekly sessions of 3000-4000 shocks with >0.44 mj/mm^2^ and V Sansone, P Romeo and V Lavanga [8] using a regimen of three sessions of 2000 shocks with 0.22-0.43 mj/mm^2^ every three weeks. Complete regression in BME was reported in 65% of participants and a reduction in 35% at six months follow-up. Complete regression was shown in all patients at one year follow-up [7]. The BME area (mm^2^) pre-ESWT was 759.98±291.39 and post-ESWT was 88.38 ± 131.32 (p<0.001) at six months follow-up [8].

The effects of ESWT was evaluated on two sport’s related musculoskeletal conditions. Y Cheng, J Zhang and Y Cai [9] reported ultrasonography changes following ESWT in 42 athletes with an established diagnosis of chronic insertional Achilles tendinopathy. Radial-ESWT with five weekly treatments of 2000 shocks at 0.16 mj/mm^2^ was used. At three months follow-up, the thickness of the Achilles tendon (mm) changed from 3.8±0.8 to 3.7±0.9 (p>0.05). The cross-sectional area (mm2) changed from 76.5±17.4 to 73.1±21.4 (p>0.05). The size of calcification (mm) changed from 8.5±8.4 to 7.2±7.2 (p<0.05). Neovascularization scores changed from 1.3±1.1 to 0.6±0.9 (p<0.05).

A total of 24 athletes with radiographic diagnosis of post-traumatic MO (ossification >1 cm in length) were evaluated following a focused-ESWT treatment regimen of three fortnightly sessions of 100 shocks per cm^2^ of ossification (0.13-0.23 mj/mm^2^).The ossification area (cm^2^) was 38.33±13.63 at baseline and 35.54±12.09 at 12 months follow-up (p=0.05) [10].

The effect of ESWT administered six weeks after arthroscopic rotator cuff repair was evaluated in 26 participants compared to 24 participants in the control group [11]. Focused-ESWT was used for one session of 1500 shocks and 0.12 mj/mm^2^ EFD. Rotator cuff integrity was evaluated with computed tomographic arthrography (stage I: anatomic healing, stage II: maintenance of insertion into the footprint with media leakage and stage III: a definite re-tear). At six month follow-up, stage I was noted in 38.5% of the ESWT and control groups, stage II in 53.8% of the ESWT group and 50% of the control group and stage III in 7.7% of the ESWT group and 16.7% in the control group.

The imaging changes of Kienbock’s disease, as characterized by isolated progressive alteration in the proximal aspect of the lunate with BME and reduced blood supply leading to collapse and severe arthritic degeneration, were evaluated using MRI. A total of 22 participants with an established diagnosis of Kienbock’s disease were treated with three, monthly focused-ESWT sessions of 4000 shocks and 0.35-0.4 mj/mm^2^. At six months follow-up, when a well-defined necrotic area was present, a significant or complete reduction of peri-lesion edema was always observed, however, necrotic area changes were not observed [12].

### Table 7 Characteristics of studies and intervention details for fracture nonunion

| **Author (year)** | **Study design** | **Condition** | **N** | **Mean age**  **±SD or (range)** | **Mean symptoms duration ±SD or (range), months** | **Area of ESWT application** | **Dosage in impulses*EFD (mJ/mm^2^)/bar** | **No. of sessions** | **Interval between sessions** | **Co- intervention, anesthesia** |
| --- | --- | --- | --- | --- | --- | --- | --- | --- | --- | --- |
| Rompe (2001) [1] | Prospective cohort | Tibial or femoral diaphyseal and metaphyseal fractures | 43 | 39.5±8.5 | >9 | Once the nonunion was localized in the ESWT focal center, the unit was docked to the skin. ESWT focus being targeted to the gap and to the adjacent cortical structures. | 3000*0.6 | 1 | NA | None, yes |
| Stojadinovic (2011) [2] | Prospective observational | Fracture nonunion at any anatomic site | 349 | 48±16 | NR | ESWT was delivered to the non-union site | 12000 (median, 4000 pulses) *0.4 | 78.2% received 1 session, the remaining received 2 sessions | NR | None, yes |
| Vulpiani (2012) [3] | Prospective cohort | Facture nonunions at any anatomic site | 143 | 41.4±15.4 | 14.1±12.9 | ESWT was constantly focused on the fracture gap and on the adjacent cortical structures with x-ray localization and in-line ultrasound. | 2500-3000*0.25-0.84.  96 patients received 1 cycle of treatment, 39 received 2 cycles, and 8 received 3 cycles at 3-month intervals | 3-5 | 2-3 days | None, only procedures performed for scaphoid nonunions were conducted using local anesthesia |
| Wang (2001) [4] | Prospective cohort | Facture nonunions of long bones | 72 | 39.4 (15-74) | >6 | Once the fracture site has been localized in position and depth, half of the impulses were applied in one plane and the other half in a different plane and the region of any metallic internal fixation such as a cortical plate is avoided. The presence of an intramedullary rod, did not interfere with the ESWT application. | 6000*0.62 for femur and tibia  3000*0.56 for humerus  2000*0.56 for radius and ulna  1000*0.47 for metatarsals. | 1, 7 patients received additional ESWT session | NA | None, yes |

### Table 8 Imaging outcome measures for fracture nonunion

| **Author (year)** | **ESWT type** | **Comparator** | **Imaging outcome** | **Follow-up** | | | |
| --- | --- | --- | --- | --- | --- | --- | --- |
|  |  |  |  | **Period** | **Baseline - F/U**  **Mean±SD** | ***P* value** | |
|  |  |  |  |  |  | **Within group** | **Between group** |
| Rompe (2001) [1] | F-ESWT | None | Standardized AP and lateral radiographs were obtained with the same exposure setting, and with a comparable positioning of the leg. A pseudarthrosis was judged healed when 4 cortices were bridged or if no gap could be detected using conventional tomography. On each radiographic evaluation at each time, 4 cortices (2on the AP radiograph, and 2 on the lateral radiograph; rarely on oblique views) were evaluated for the amount of cortical bridging. | 8 weeks then monthly  thereafter until 9 months or until adequate bony healing was determined | A success rate of 50% of 8 tibial and 66% of 9 femoral post-fracture nonunions was achieved. Regarding postosteotomy pseudarthrosis, consolidation occurred in 82% of 11 tibias and 80% of 15 femurs. | NR | NA |
| Stojadinovic (2011) [2] | F-ESWT | None | Reestablishment of cortical continuity of a minimum of 3 of 4 cortices defined fracture-healing. Stress radiography and/or CT scans were obtained if the adequacy of fracture-healing could not be assessed with radiography. Imaging assessment included anteroposterior and lateral radiographs. | 1,3,6 months | 282 (80.8%) healed while 67(19.2%) persisted following ESWT | NR | NA |
| Vulpiani (2012) [3] | F-ESWT | None | A nonunion was deemed healed when 4 cortices (2 on the anteroposterior radiograph and 2 on the lateral radiograph) were bridged or if no gap could be detected using conventional tomography. Fractures were determined to be either completely healed (i.e., bridging callus on 4 cortices), partially healed (i.e., incomplete bone callus formation, not requiring further treatment) or not healed. | 3,6,9,12 months | 80 of the 143 nonunions (55.9%) ultimately healed after ESWT at an average time of 7.6±16.3 months, 41 were partially healed (28.7%), and 22 had no healing (15.4%). | NR | NA |
| Wang (2001) [4] | F-ESWT | None | Radiographs were used to assess alignment, callus formation, the maximal and minimal fracture gaps and the presence of bony union across the fracture site. Tomography was done for patients where adequate information could not be obtained with radiographs | 6 weeks, 3,6,9,12 months | The mean minimal fracture gap (mm) pre-ESWT was 1.87 and 1.23 post-ESWT. Consolidation was shown in 28 (40%) and non-apparent change in 42 (60%). There was union in 19/38 (50%) with hypertrophic non-union, 9/19 (47.4%) with non-union with a defect and 0/13 with atrophic non-union | =0.002 for fracture gap | NA |
| NA: not applicable, NR: not reported | | | | | | | |

**REFERENCES**

1. Rompe JD, Rosendahl T, Schollner C, Theis C: **High-energy extracorporeal shock wave treatment of nonunions**. *Clinical Orthopaedics and Related Research* 2001(387):102-111.

2. Stojadinovic A, Kyle Potter B, Eberhardt J, Shawen SB, Andersen RC, Forsberg JA, Shwery C, Ester EA, Schaden W: **Development of a prognostic naive bayesian classifier for successful treatment of nonunions**. *The Journal of bone and joint surgery American volume* 2011, **93**(2):187-194.

3. Vulpiani MC, Vetrano M, Conforti F, Minutolo L, Trischitta D, Furia JP, Ferretti A: **Effects of extracorporeal shock wave therapy on fracture nonunions**. *American journal of orthopedics (Belle Mead, NJ)* 2012, **41**(9):E122-127.

4. Wang CJ, Chen HS, Chen CE, Yang KD: **Treatment of nonunions of long bone fractures with shock waves**. *Clinical Orthopaedics and Related Research* 2001(387):95-101.

5. Cho NS, Park YH, Hwang JH, Yoon YC, Park MJ, Yoo JC, Park WH: **Ultrasonographic features of the tennis elbow: Which is more responsive for extracorporeal shock wave therapy?** *Journal of Musculoskeletal Pain* 2012, **20**(2):100-106.

6. Gunduz R, Malas FU, Borman P, Kocaoglu S, Ozcakar L: **Physical therapy, corticosteroid injection, and extracorporeal shock wave treatment in lateral epicondylitis: Clinical and ultrasonographical comparison**. *Clinical Rheumatology* 2012, **31**(5):807-812.

7. Gao F, Sun W, Li Z, Guo W, Kush N, Ozaki K: **Intractable bone marrow edema syndrome of the hip**. *Orthopedics* 2015, **38**(4):e263-270.

8. Sansone V, Romeo P, Lavanga V: **Extracorporeal Shock Wave Therapy Is Effective in the Treatment of Bone Marrow Edema of the Medial Compartment of the Knee: A Comparative Study**. *Medical Principles and Practice* 2017, **26**(1):23-29.

9. Cheng Y, Zhang J, Cai Y: **Utility of Ultrasonography in Assessing the Effectiveness of Extracorporeal Shock Wave Therapy in Insertional Achilles Tendinopathy**. *BioMed Research International* 2016, **2016**:2580969.

10. Buselli P, Coco V, Notarnicola A, Messina S, Saggini R, Tafuri S, Moretti L, Moretti B: **Shock Waves in the Treatment of Post-Traumatic Myositis Ossificans**. *Ultrasound in Medicine and Biology* 2010, **36**(3):397-409.

11. Kim JY, Lee JS, Park CW: **Extracorporeal shock wave therapy is not useful after arthroscopic rotator cuff repair**. *Knee Surgery, Sports Traumatology, Arthroscopy* 2012, **20**(12):2567-2572.

12. D'Agostino C, Romeo P, Amelio E, Sansone V: **Effectiveness of ESWT in the Treatment of Kienbock's Disease**. *Ultrasound in Medicine and Biology* 2011, **37**(9):1452-1456.
